# GmMEKK2 Disrupts the MKK1/2–MPK4 Cascade to Amplify Immune Signalling and Confer Enhanced Resistance to Soybean Mosaic Virus

**DOI:** 10.1111/mpp.70184

**Published:** 2025-11-30

**Authors:** Xuanbo Zhong, Jingxiang Luo, Yucheng Ruan, Longlong Hu, Yue Shu, Guixiang Tang

**Affiliations:** ^1^ Zhejiang Provincial Key Laboratory of Crop Genetic Resources, Institute of Crop Science Zhejiang University Hangzhou Zhejiang China; ^2^ China Tobacco Fujian Industry Co. Ltd. Xiamen Fujian China

**Keywords:** *GmMEKK2*, immune response, MAPK cascade, ROS homeostasis, salicylic acid, SMV

## Abstract

Mitogen‐activated protein kinase kinase kinase (MAPKKK) assumes a pivotal position within the MAPK cascade, converting external stimuli into intracellular responses and mediating plant stress resistance. However, there are limited reports investigating its function in regulating resistance to soybean mosaic virus (SMV). Here, a *MAPKKK2‐like* gene *GmMEKK2* (LOC100798607) was identified from an SMV‐resistant cultivar, with an amino acid sequence containing the typical conserved G(T/S)Px(W/Y/F)MAPExV domain, showing homology to AtMEKK1, AtMEKK2 (SUMM1) and OsMAPKKK5. Overexpression of *GmMEKK2* in soybean reduced SMV accumulation and the disease index, mitigated the yield loss after SMV inoculation and improved yield traits. In contrast, silencing *GmMEKK2* via virus‐induced gene silencing (VIGS) significantly enhanced SMV susceptibility, with increased disease index and viral content. Transcriptionally, defence responce genes such as *PR1*s and *RPM1*s were pre‐activated in the overexpression lines before SMV infection, conferring soybean with preventive resistance. While non‐transgenic plants showed severe down‐regulation of basic metabolic genes and strong induction of genomic repair genes at 14 days post‐inoculation (dpi), overexpression lines exhibited minimal changes. Despite lacking kinase activity, GmMEKK2 bound with GmMKK1 and GmMPK4A to block the MKK1/2–MPK4 cascade, thereby indirectly enhancing the salicylic acid‐induced defence responses. Additionally, *GmMEKK2* overexpression elevated basal reactive oxygen species (ROS) levels to trigger autoimmunity, while maintaining ROS homeostasis via the antioxidant enzyme system in soybean. This study clarifies the function and molecular mechanism of *GmMEKK2* in SMV resistance, providing a strategy for improving soybean SMV resistance.

## Graphical abstract

Overexpressing *GmMEKK2* in soybean ( *Glycine max* ) blocks the MEKK1‐MKK1‐MPK4 cascade via protein interaction, triggering defence responses. An increase in reactive oxygen species levels and elevated expression of defence genes such as *PR1* leads the plant to exhibit an autoimmune state, enhancing resistance to SMV. Thus, *GmMEKK2* is a promising target for soybean breeding.

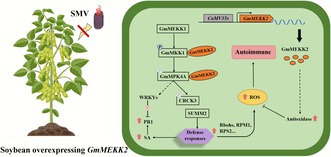

## 1 Introduction

Soybean mosaic virus (SMV) disease is a prevalent plant disease worldwide. It causes an average annual soybean ( *Glycine max* ) yield loss of approximately 25%–50%, reaching more than 86% in some production areas (Song et al. 2016; Whitham et al. 2016). The pathogen is a single‐stranded, positive‐sense RNA virus that belongs to the genus *Potyvirus*. Several SMV strains, including G1–G7 and SC1–SC21, have been identified from a range of susceptible soybean cultivars since Clinton discovered SMV in the legume in 1915 (Cho and Goodman 1979; Li et al. 2010; Ma et al. 2016). Plants infected with SMV experience chloroplast membrane damage and thylakoid vacuolation, resulting in decreased photosynthetic efficiency (Hartman et al. 2016). SMV infections also cause seed mottle and seriously affect seed vigour and quality (Jossey et al. 2013). The extent of damage mainly depends on the plant genotype and the virus strain (Shakiba et al. 2013; Usovsky et al. 2022). In recent decades, the functions of the *Rsv1*, *Rsv3*, *Rsv4* and *Rsv5* SMV resistance loci, which are present on chromosomes Gm02, Gm13 and Gm14, have been investigated (Klepadlo et al. 2017a, 2017b). *R* gene pyramiding is an excellent strategy to breed SMV‐resistant plants (Shi et al. 2009). However, due to the lack of pleiotropic interactions between *R* genes and the different novel virus strains that are constantly emerging (Xiang et al. 1991), it is difficult to prevent or control SMV effectively (Widyasari et al. 2020). Therefore, it is necessary to explore potential resistance genes and breed novel SMV‐resistant soybean cultivars by modern biotechnology.

Mitogen‐activated protein kinase kinase kinases (MAPKKKs) are at the start of the MAPK cascade and participate in the regulation of complex signal networks (Zhu et al. 2022). They play vital roles in plant responses to abiotic and biotic stresses, such as high temperature, drought, salinity and pathogens (Ning et al. 2010; McNeece et al. 2019; Su et al. 2020; Gao et al. 2022). In the canonical MAPK cascade model, signals originating from stimulated plasma membrane receptors or sensors are first received by MAPKKKs (also known as MAP3Ks or MEKKs). Then, the intracellular signals are relayed and amplified to downstream components through the reversible three levels of MAPKKKs‐MAPKKs‐MAPKs phosphorylation (Rasmussen et al. 2012) or through a MAPKKs‐MAPKs protein complex bound by MAPKKKs (Suarez‐Rodriguez et al. 2007). By sequentially altering the activity of substrate proteins such as transcription factors and ribosomal proteins, a wide array of responses, including changes in gene expression, are initiated (Rodriguez et al. 2010). Interestingly, MAPKKKs can directly regulate downstream target proteins, bypassing intermediate cascade components (Miao et al. 2007).

Multiple studies support central roles for MAPK cascades in pathogen defence in *Arabidopsis thaliana* , tobacco, rice and tomato (Yang et al. 2001; Zhang and Klessig 2001; Chen et al. 2021; Wang, Wei, et al. 2023). Crosstalk exists between the MAPK cascade and other pathways in response to pathogens and other stress‐induced signalling pathways. Different cascade pathways can share the same components, ensuring substrate specificity and resulting in more efficient transduction. Activated MAPKKKs are positively or negatively involved in the regulation of defence phenotypes, such as dwarfism, the accumulation of reactive oxygen species (ROS) and the induction of pathogenesis‐related (PR) gene expression when plants are infected with pathogens (Jammes et al. 2009; Pitzschke et al. 2009; Xu et al. 2018). *MEKK1* has been extensively studied in *Arabidopsis* (Petersen et al. 2000; Gao et al. 2008; Qiu et al. 2008). The MEKK1‐MKK1/MKK2‐MPK4 module negatively regulates defence responses; *mekk1*, *mkk1/2* and *mpk4* mutants all show similar constitutive defence responses (Petersen et al. 2000; Gao et al. 2008; Qiu et al. 2008). The kinase‐deficient form of *Enhanced Disease Resistance 1* (*EDR1*) is a homologue of *MEKK1* and negatively regulates salicylic acid (SA)‐inducible defence responses. The overexpression of *EDR1* enhances powdery mildew resistance and ethylene‐induced senescence (Tang and Innes 2002). Similar regulatory patterns for pathogen infection have also been found in *MEKK1* homologues in other plants (Xu et al. 2018). *Nicotiana Protein Kinase 1* (*NPK1*), a *MAPKKK* gene in tobacco, interferes with the functions of the disease‐resistance genes *N*, *Bs2* and *Rx*. *NPK1*‐silenced plants exhibit reduced cell sizes and an overall dwarf phenotype (Jin et al. 2002). In contrast, Asai et al. found that the MEKK1‐MKK4/5‐MPK3/6‐WRKY22/WRKY29 signalling module acts as downstream of the flagellin receptor, inducing the expression of *PR* genes to enhance resistance to bacterial and fungal pathogens in *Arabidopsis* (Asai et al. 2002). *Botrytis cinerea* infections can also lead to the rapid transcriptional induction of *MAP3K18*, *MAP3K19* and *MAP3K20* (Menges et al. 2008).

Current research on *MAPKKKs* is mostly focused on homologues of *MEKK1* and their associated downstream components in model plants. Limited studies have been conducted on other members of the *MAPKKK* gene family, particularly *MEKK2*, a tandemly duplicated gene of *MEKK1*. In *Arabidopsis*, *MEKK2* acts downstream of the MEKK1‐MKK1/MKK2‐MPK4 module. It positively regulates the defence response by increasing expression abundance when the components of this cascade are disrupted (Kong et al. 2012; Su et al. 2013). A *MAPKKK 2‐like* sequence, *GmMEKK2* (LOC100798607), in soybean has been cloned based on microarray library data of differential gene expression between a resistant cultivar Ji‐75 and a susceptible cultivar Changnong‐15 after SMV inoculation (He 2011). However, its specific function and role in SMV resistance remain unknown. In this study, plants overexpressing *GmMEKK2* were generated using an *Agrobacterium tumefaciens* ‐mediated transformation method in soybean. The *GmMEKK2* function was demonstrated, and the potential molecular mechanisms of *GmMEKK2* against SMV were investigated using comparative transcriptomics. This study provides novel evidence and strategies for the use of *GmMEKK2* to improve SMV resistance in soybean.

## 2 Results

### 2.1 Phylogenetic Analysis and Sequence Alignment of *GmMEKK2*

*GmMEKK2* is a homologue of *MAPKKK2*, which was previously reported as a candidate SMV strain SC7 resistance gene in soybean (He 2011). The coding sequence (CDS) of *GmMAPKKK* (LOC100798607) is 1026 bp and encodes a protein of 341 amino acids. *GmMEKK2* exhibits a close phylogenetic relationship with *MAPKKK17* in legume species such as *Glycine soja* , *Cicer arietinum* and *Medicago truncatula* (Figure S1A). *GmMEKK2* shares a high sequence similarity with *GmMEKK1*, *GmMAPKKK5‐like* and their homologous genes in *Arabidopsis* and rice, including *AtMEKK1*, *AtMEKK2* (*SUMM1*) and *OsMAPKKK5*. These genes play pivotal roles in plant immunity. The sequence alignment also indicated that the sequence similarity among legume MAPKKK proteins is 63.4%, and GmMEKK2 contains the conserved MAPKKK signature motif G(T/S)Px(W/Y/F) MAPExV (Wang et al. 2016) when compared with the homologous sequences in the other five species (Figure S1B).

### 2.2 Overexpressing *GmMEKK2* Enhanced Soybean SMV Resistance

To investigate the function of *GmMEKK2*, its CDS, under the control of the CaMV 35S promoter, was cloned into a plant binary expression vector that contained the *bar* gene as a selective marker (Figure S2A). As shown in Table S2, 17 putative transgenic seedlings were grown from 434 explants via three batches of *A. tumefaciens*‐mediated transformations (Figure S3). Seven independent transgenic lines overexpressing *GmMEKK2* were identified by Basta selection, protein quick dip strips, and PCR (Figure S2B–D). The relative expression levels of *GmMEKK2* in Lines 1, 3, 6 and 7 were found to be 2.4–4.5 times higher than in nontransgenic (NT) plants (Figure S2E). A Southern blot analysis (Figure S2F) demonstrated a single copy of exogenous DNA fragments containing the *GmMEKK2* gene integrated into the genome in Lines 1, 3, 6 and 7.

**FIGURE 1:**
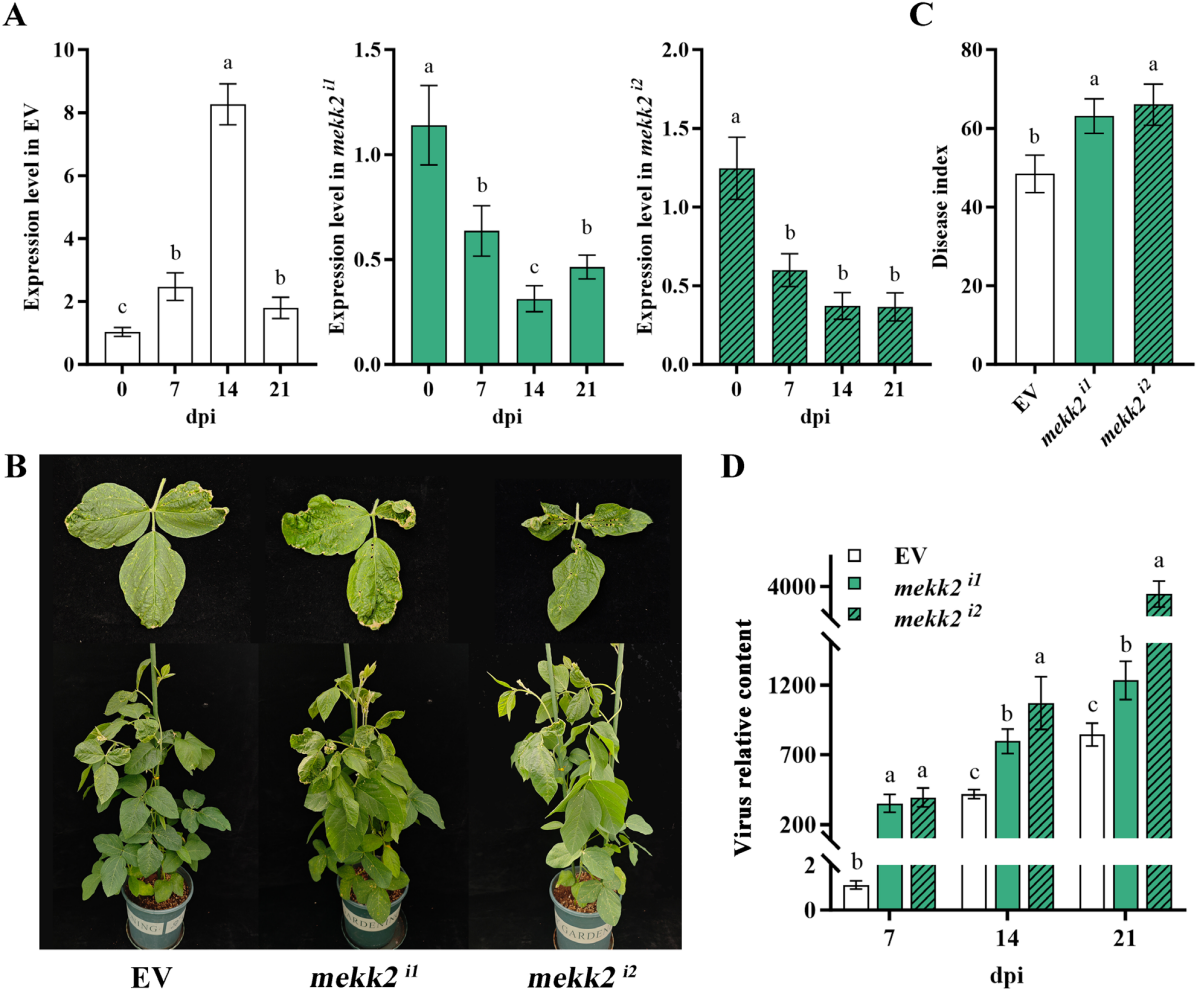
Silencing of *GmMEKK2* by virus‐induced gene silencing (VIGS) increased soybean mosaic virus (SMV) susceptibility. (A) Efficiency of *GmMEKK2* silencing in empty vector control (EV) and *GmMEKK2*‐silenced *mekk2* ^*i1*^ and *mekk2* ^*i2*^ plants at 0, 7, 14 and 21 days post‐inoculation (dpi). (B) Phenotypes of soybean after SMV infection: EV and *GmMEKK2*‐silenced lines generated using VIGS. Images were taken at 21 dpi. (C) Disease indices of plants at 21 dpi. Lowercase letters denote statistically significant differences among groups at the same time point (*p* < 0.05, one‐way ANOVA with Duncan's test). (D) Relative SMV accumulation in top new leaves of EV and *GmMEKK2*‐silenced plants at 7, 14 and 21 dpi, quantified by reverse transcription‐quantitative PCR using SMV coat protein‐specific primers.

In addition, two sequences within kinase domains were specifically targeted to silence *GmMEKK2* expression in soybean using tobacco rattle virus (TRV)‐based virus‐induced gene silencing (VIGS), achieving 56%–70% knockdown efficiency (Figure 1A).

**FIGURE 2:**
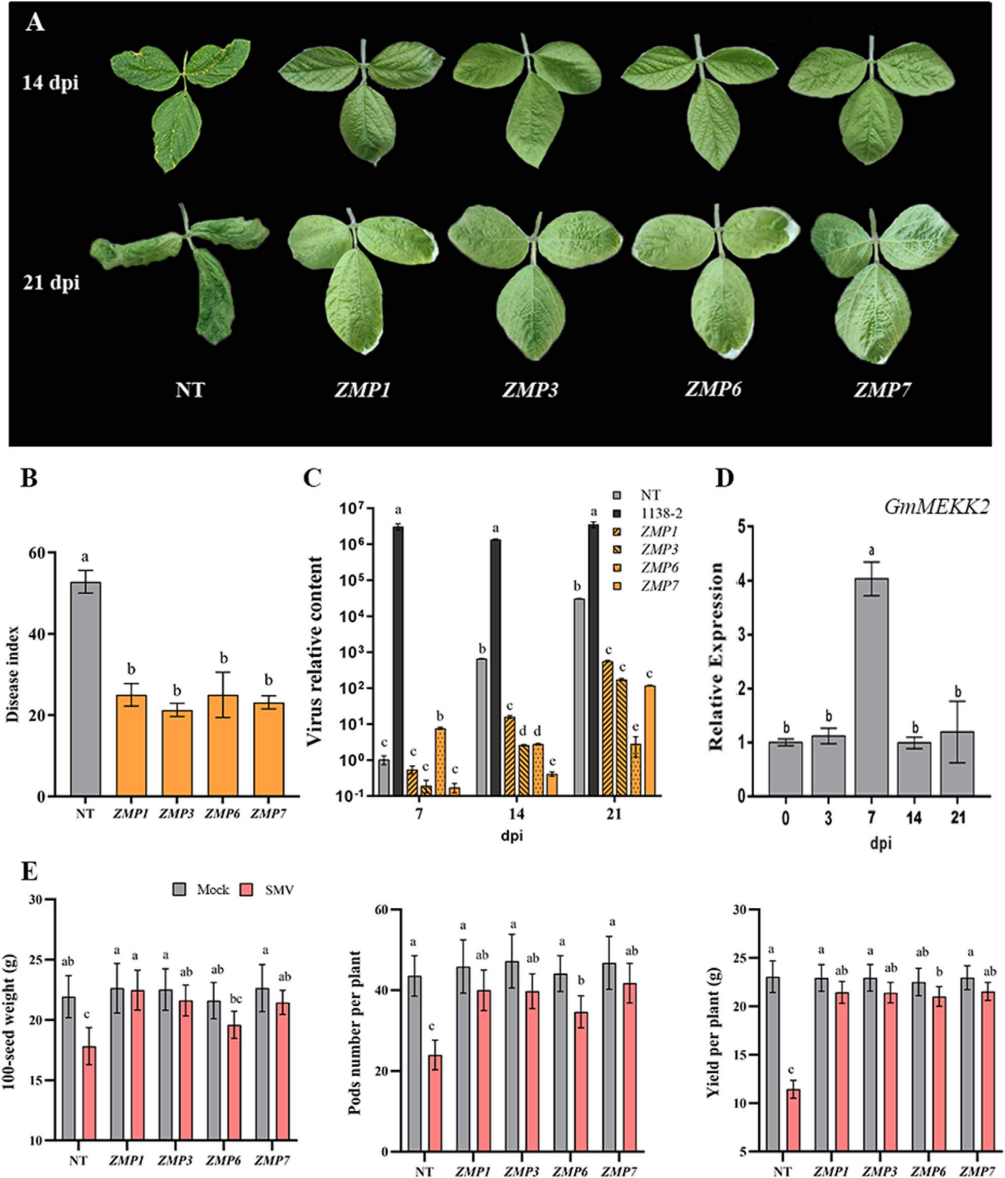
Overexpression of *GmMEKK2* improved soybean mosaic virus (SMV) resistance in soybean. (A) Infection symptoms on soybean leaves after SMV inoculation. NT, nontransgenic plants; ZMP1, 3, 6 and 7 indicate *GmMEKK2*‐overexpression lines 1, 3, 6 and 7, respectively. (B) Disease indices of NT and each *GmMEKK2*‐overexpression line. The disease index was investigated at 21 days post‐SMV‐inoculation. (C) Quantification of SMV content in soybean leaves. SMV‐susceptible line 1138‐2 was used as a positive control. (D) The *GmMEKK2* expression pattern in NT plants after SMV inoculation. (E) Comparison of yield traits between NT and overexpression plants after SMV infection. Mock‐inoculated plants served as the control. Values labelled with different lowercase letters (a–e) are significantly different at *p* < 0.05 as determined by Duncan's test.

SMV inoculations were performed by rubbing to investigate *GmMEKK2's* function in transgenic plants, including overexpression lines ZMP1, ZMP3, ZMP6, ZMP7 and silenced lines *mekk2* ^*i1*^ and *mekk2* ^*i2*^, while NT as well as empty vector (EV) plants were used as controls. Overexpression of *GmMEKK2* generally enhanced resistance to SMV, although the degree of improvement varied among different strains (Figure S4). The resistance to SMV strain SC7 was significantly improved, followed by SC5 and SC8, while the resistance against SC3 was relatively weak. Specifically, the disease index (DI) (Figure S4A) and viral content (Figure S4B) of the overexpression lines inoculated with SC3, SC5 and SC8 were all higher than those inoculated with SC7. A more detailed characterisation of resistance to SC7 showed that leaves of overexpression lines showed slight shrinkage without mosaic symptoms at 14 days post‐inoculation (dpi), whereas leaves of NT showed obvious shrinkage and slight mosaic mottling. At 21 dpi, the leaves of the overexpression lines showed mild symptoms, while some leaves of NT plants were deformed with bleb‐like bulges and large areas of yellow mottling (Figure 2A). Compared with EV controls, *GmMEKK2*‐silenced *mekk2* ^*i1*^ and *mekk2* ^*i2*^ plants exhibited significantly enhanced leaf crinkling phenotypes (Figure 1B). The DI evaluation revealed distinct resistance levels among genotypes (Figures 2B and 1C). Overexpression lines exhibited significantly reduced DI values, ranging from 19.4 to 30.6, which were classed as moderate resistance (MR). In contrast, silenced lines displayed hypersusceptibility, with DI values exceeding 60 and were thus categorised as susceptible (S). NT and EV plants showed comparable moderate susceptibility (MS), with DI values of 52.8 and 48.5, respectively. A double‐antibody sandwich (DAS)‐ELISA showed that the sample:negative control absorbance value ratios of all the transgenic lines were below 1, whereas the ratio was as high as 2.36 in NT plants (Table S3). This was consistent with the symptomology. The quantitative virus results revealed a significantly higher virus content in the susceptible cultivar 1138‐2, which persisted from 7 to 21 dpi and reached a level 10^6^ times that of the control. The virus content in NT leaves increased and exceeded 10^4^ at 21 dpi (Figure 2C). Despite the increase during infection, soybean overexpressing *GmMEKK2* had significantly lower viral accumulations than the NT at all time points. In addition, compared with EV controls, the silenced plants showed significantly elevated viral accumulations (Figure 1D). For yield traits, SMV infection caused a severe yield loss in NT plants, with significant reductions in seed weight, pod number and individual plant yield, which decreased to 11.43 g per plant (Figure 2E). However, the overexpression of *GmMEKK2* effectively alleviated yield losses, with the yield of the ZMP1, ZMP3 and ZMP7 lines being almost unaffected. In addition, SMV induced a notable increase in *GmMEKK2* expression at 7 dpi, followed by a subsequent return to a normal level, in the NT (Figure 2D). The ZMP3 line was selected for subsequent transcriptome analysis because it had the highest *GmMEKK2* expression levels and the mildest symptoms after SMV inoculation.

### 2.3 Overexpressing *GmMEKK2* Mitigated the Impact of SMV at the Transcriptional Level

The leaves of NT and ZMP overexpression lines at 0, 7 and 14 dpi were sampled for transcriptome analyses. After removing the low‐quality data, over 40 million clean reads for each library (Table S4) were mapped to the soybean reference Genome (Wm82a2.v1), with 47,259 transcripts identified from all the samples. NT_7d (7 dpi), ZMP_CK (uninoculated) and ZMP_7d clustered together in the principal component analysis (PCA), indicating that their transcript profiles were similar. In contrast, the transcript profiles of NT_CK, NT_14d and ZMP_14d were quite different, as shown in Figure S5. The expression levels of nine randomly selected genes at each stage of SMV infection were measured by reverse transcription‐quantitative PCR (RT‐qPCR) (Figure S6), and the results were highly related to RNA‐Seq data.

After the SMV inoculation, there were 2959, 8077 and 11,110 differentially expressed genes (DEGs) identified in the comparisons NT_7d versus NT_CK, NT_14d versus NT_CK and NT_14d versus NT_7d, respectively (Figure S7A). Most of these DEGs were down‐regulated at 7 dpi, while the majority of up‐regulated DEGs occurred by 14 dpi (Figure S7D). The overexpression of *GmMEKK2* alleviated the impact of the virus to some extent as only 284, 1499 and 491 DEGs, respectively, were identified in the three comparison groups of ZMP plants (Figure S7B). DEGs involved in basal metabolism, such as carbohydrate metabolism, nitrogen metabolism, and photosynthesis, were significantly down‐regulated in NT plants after SMV inoculation. Compared with NT_CK, the expression levels of genes encoding α‐glucan phosphorylase, β‐amylase and phosphoglucomutase decreased to 0.61, 0.73 and 0.48‐fold, respectively, at 7 dpi and to 0.26, 0.45, 0.50‐fold, respectively, at 14 dpi. Similarly, genes encoding nitrate reductase, glutamine synthetase and high‐affinity nitrate transporter were down‐regulated to approximately 0.3‐fold at 14 dpi. Some important signalling molecules in the MAPK cascade and plant–pathogen interaction pathways were also suppressed by SMV infection in NT plants, such as MAPKKK18, PP2C, PR1s and calmodulins (Table S5). However, the expression levels of these genes in ZMP plants were not greatly changed.

DEGs such as *MAPK1/3*, *MKS1*, *WRKY39*, *WRKY5*, *ERF1* and *ChiB* in the MAPK signalling pathway and plant hormone signal transduction pathway were strongly induced at 7 dpi in NT plants. In addition, the expression levels of DEGs related to the hypersensitive response, such as *Rboh*s and *RPM1*s, were significantly increased. Three *Rboh* genes were up‐regulated 3.99, 2.73 and 5.23‐fold at 7 dpi. In flavonoid and isoflavone biosynthesis pathways, genes associated with biotic stress resistance that encoded caffeoyl‐CoA O‐methyltransferase, chalcone isomerase and chalcone synthase were also up‐regulated to varying degrees (Table S6). As the SMV infection strengthened, the overall gene expression pattern in NT plants changed drastically. There were over 5044 up‐regulated DEGs in NT plants at 14 dpi (Figure S7D). The expressions of mini‐chromosome maintenance genes, *MCM*s, were up‐regulated to 3.7–9.5‐fold at 14 dpi. This may represent an adaptive strategy to cope with severe viral infections and avoid DNA replication‐related stress and cell death. Moreover, genes involved in DNA damage repair, including *PCNA*, *MSH7* and *BRCA2*, and genes participating in DNA damage responses, such as *BRCT*s, were also significantly induced (Table S7). These DEGs were mainly enriched in the MAPK signalling (gmx04016), DNA replication (gmx03030), mismatch repair (gmx03430), homologous recombination (gmx03440) pathways (Figure S8). In contrast to the substantial alterations in the transcriptome of NT, only a small fraction of genes in ZMP plants exhibited a significant expression change (Figure S7). This is consistent with the mild phenotypic changes in ZMP plants after SMV inoculation.

### 2.4 *GmMEKK2* Triggered Autoimmunity by Blocking MKK1/2–MPK4 Signalling

**FIGURE 3:**
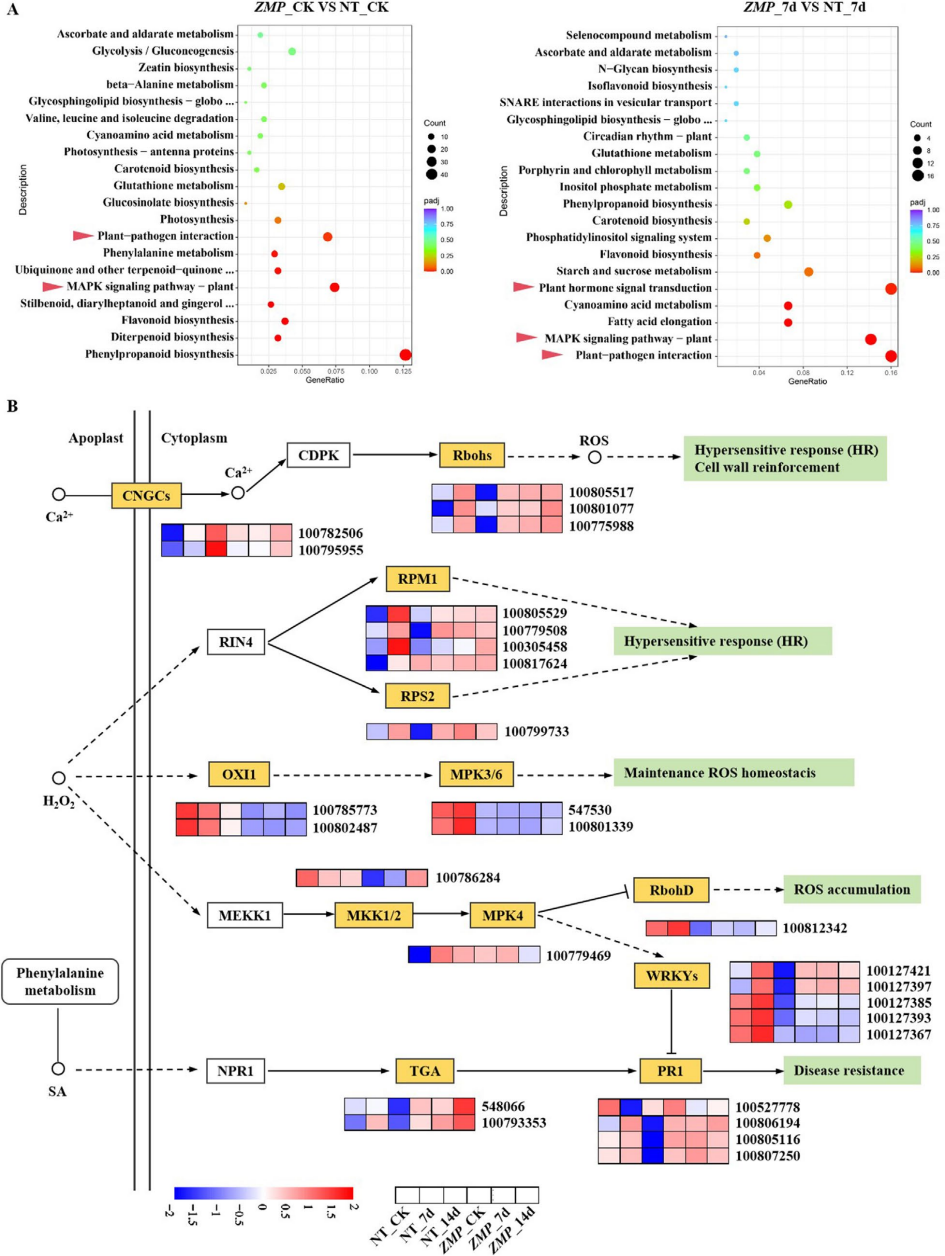
Expression profiles of key differentially expressed genes (DEGs) between nontransgenic (NT) and *GmMEKK2*‐overexpression lines (ZMP) involved in the reactive oxygen species (ROS)‐ and salicylic acid (SA)‐related pathways. (A) KEGG enrichment analysis of DEGs between NT and ZMP plants. Left: NT_CK versus ZMP_CK (uninfected controls); Right: NT_7d versus ZMP_7d (7 days post‐SMV‐inoculation [dpi]). Points represent enriched pathways, with size indicating gene count and colour reflecting −log_10_(adjusted *p*‐value). Red arrows highlight defence‐related pathways. (B) Expression dynamics of key components among MAPK, plant hormone signalling and plant–pathogen interaction pathways. Schematic depicts signal transduction from apoplast to cytoplasm, including Ca^2+^ sensors (CNGCs and CDPKs), ROS producers (Rbohs) and SA‐induced defence protein (PR1). Heatmaps show expression levels across conditions (NT and ZMP at 0, 7 and 14 dpi), with gene IDs labelled.

**FIGURE 4:**
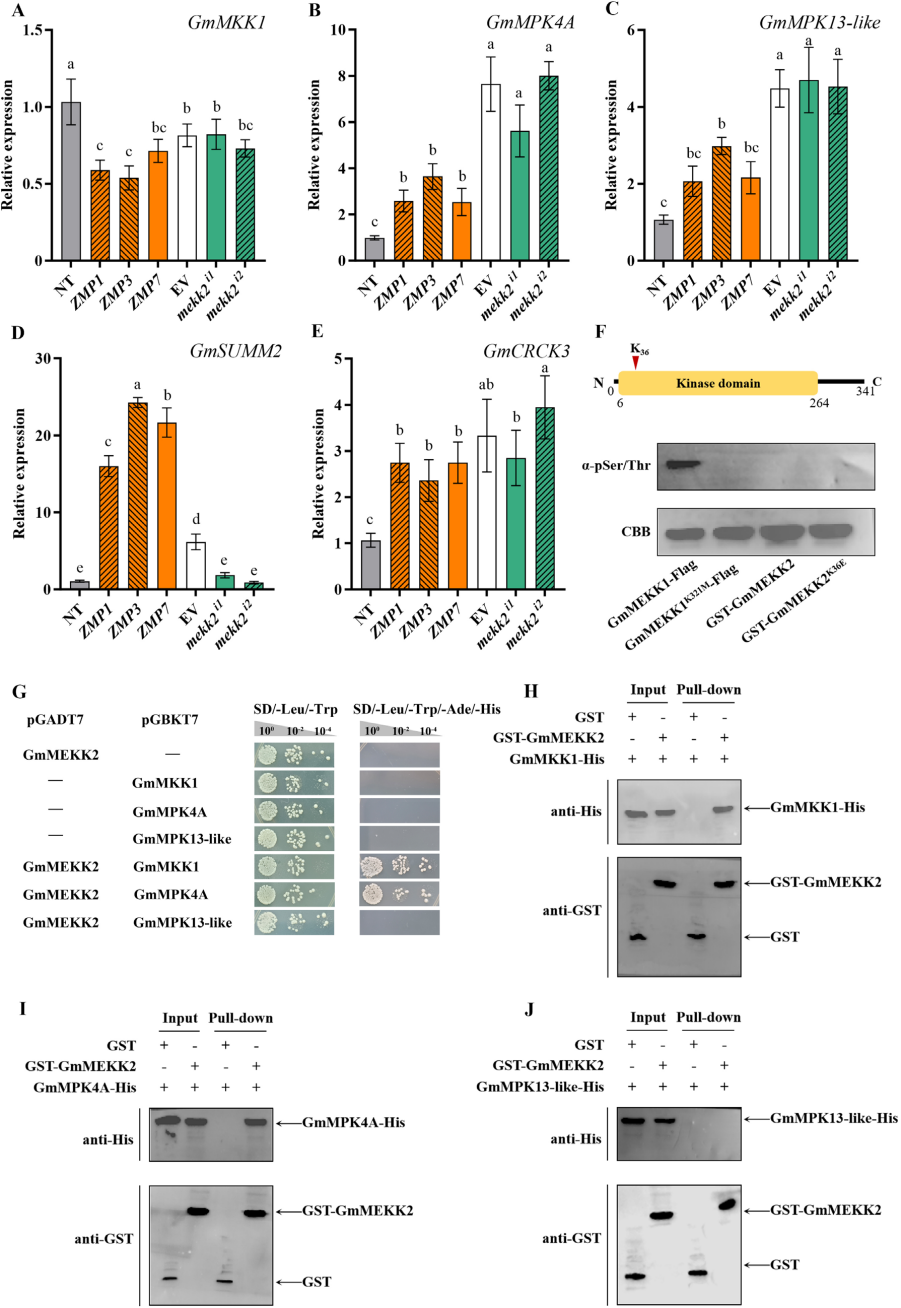
Kinase activity of GmMEKK2 is dispensable for its function in mediating defence signalling. (A–E) Relative expression levels of (A) *GmMKK1*, (B) *GmMPK4A*, (C) *GmMPK13‐like*, (D) *GmSUMM2* and (E) *GmCRCK3* in nontransgenic control (NT), *GmMEKK2*‐overexpression lines (ZMP1, ZMP3 and ZMP7), empty vector control (EV) and *GmMEKK2‐*silenced lines (*mekk2* ^*i1*^ and *mekk2* ^*i2*^). Lowercase letters denote significant differences at *p* < 0.05 as determined by one‐way ANOVA with Duncan's test. (F) Domain architecture of GmMEKK2 highlighting the kinase domain (6–264 amino acids) and ATP‐binding site (K36). Autophosphorylation of GmMEKK2 was assessed by immunoblotting with α‐pSer/Thr antibody. Recombinant proteins GmMEKK1‐FLAG and GmMEKK1^K321M^‐FLAG were used as positive and negative controls, respectively. Coomassie brilliant blue staining validated the equal loading of recombinant proteins. (G) Yeast two‐hybrid analysis of GmMEKK2 interaction with GmMKK1, GmMPK4A and GmMPK13‐like. Transformants expressing pGADT7 and pGBKT7 constructs were grown on SD/−Leu/−Trp (control) and SD/−Leu/−Trp/−Ade/−His (selection) media. (H–J) Glutathione S‐transferase (GST) pull‐down assays with anti‐His and anti‐GST antibodies demonstrating direct binding between GST‐GmMEKK2 and (H) GmMKK1‐His, (I) GmMPK4A‐His and (J) GmMPK13‐like‐His.

The DEGs between NT and ZMP plants further revealed the mechanisms responsible for immune activation in *GmMEKK2*‐overexpressing plants. In total, 2363, 788 and 9875 DEGs were identified in ZMP_CK versus NT_CK, ZMP_7d versus NT_7d and ZMP_14d versus NT_14d, respectively (Figure S7C). Compared with NT_CK, the MAPK signalling pathway, plant–pathogen interaction pathway and phenylpropanoid metabolism were activated in ZMP_CK, but photosynthesis and diterpenoid biosynthesis were inhibited (Figure 3A). Additionally, there was a relatively close clustering relationship between ZMP_CK and NT_7d (Figure S7E). The common DEGs of the three comparison groups between ZMP and NT plants were analysed (Table S8). *GmMEKK2* overexpression appeared to modulate the transcription levels of the MEKK1–MKK1/2–MPK4 cascade and downstream defence genes (Figure 3B). In unchallenged plants, *GmMKK1* expression was significantly suppressed in overexpression lines, whereas *GmSUMM2*, and *GmCRCK3* were up‐regulated (Figure 4A–E). In silenced lines, *GmSUMM2* was significantly down‐regulated, whereas other genes' expression levels remained unchanged. Phosphorylation assays were conducted on the GmMEKK2 kinase, and the results indicated that the recombinant protein GST‐GmMEKK2 lacked autophosphorylation activity (Figure 4F). However, yeast two‐hybrid (Y2H) (Figure 4G) and pull‐down assays (Figure 4H,I) demonstrated that GmMEKK2 bound with GmMKK1 and GmMPK4A in vivo and in vitro, respectively, indicating that GmMEKK2 could block the cascade signal transduction, thereby activating autoimmunity. Nevertheless, no interaction was detected between GmMEKK2 and GmMPK13‐like, which is a homologue of GmMPK4A (Figure 4J).

### 2.5 *GmMEKK2* Promoted SA‐Induced Immune Responses

**FIGURE 5:**
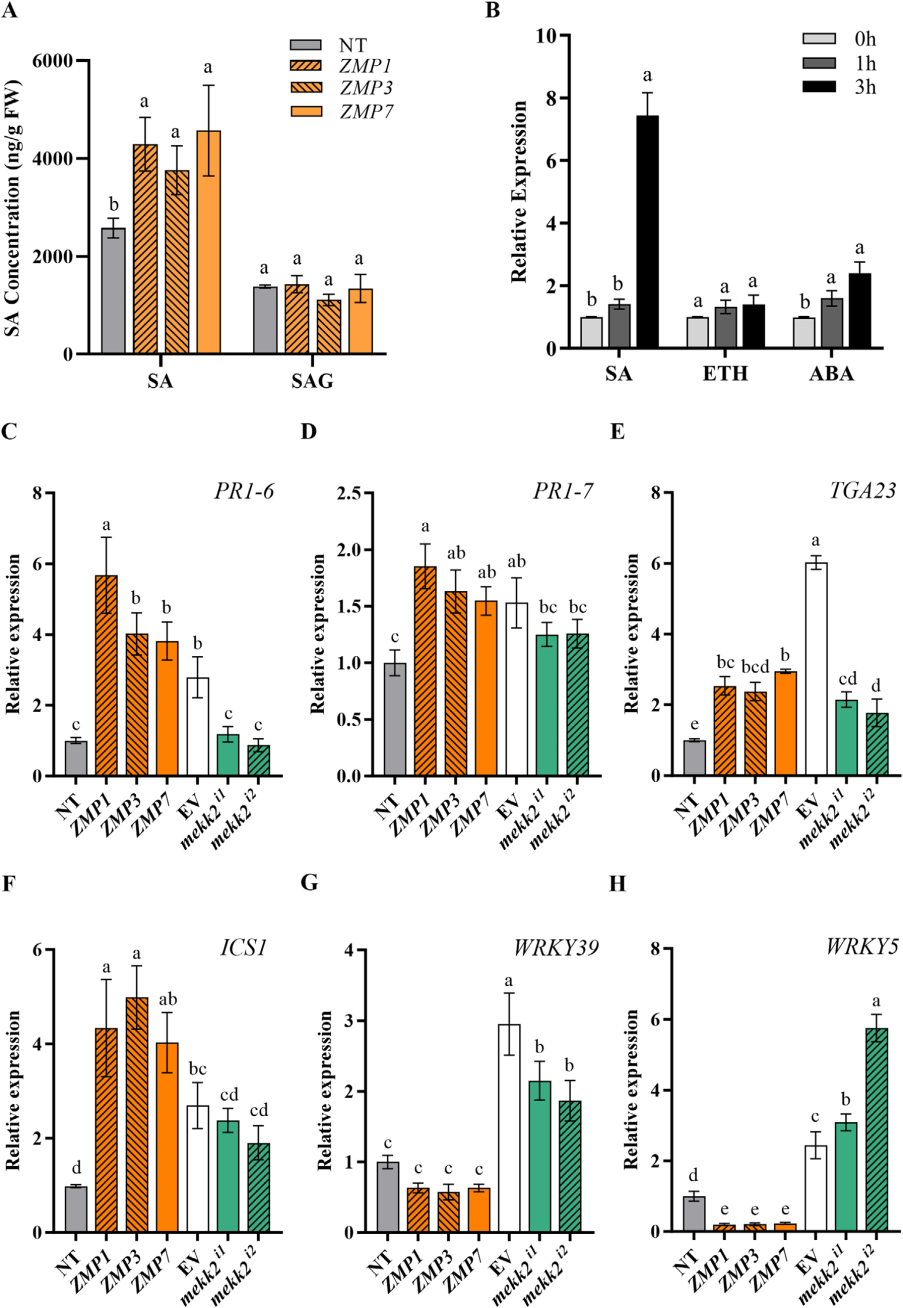
*GmMEKK2* promotes the immune response induced by salicylic acid (SA). (A) Contents of free (SA) and bound salicylic acid (SAG) in nontransgenic (NT) and *GmMEKK2*‐overexpression (ZMP) lines. (B) *GmMEKK2* expression in NT plants after exogenous hormone treatments. ETH, ethylene; ABA, abscisic acid (C–H) Expression of pivotal genes in the SA signalling pathway in NT, *GmMEKK2*‐overexpression and *GmMEKK2*‐silenced (*mekk2^i1^* and *mekk2^i2^*) plants at 7 days post‐inoculation. EV, empty vector. Values labelled with different lowercase letters (a–c) are significantly different at *p* < 0.05 as determined by Duncan's test.

DEGs between NT and ZMP plants were also enriched in the plant hormone transduction pathway. Key genes in the salicylic acid (SA) signalling pathway, such as *NPR1* and *TGA*, and the disease resistance gene *PR1* were all significantly up‐regulated in ZMP plants (Figure 3B). The overexpression of *GmMEKK2* partially alleviated the expression inhibition of the *PR1* gene through the MKK1/2–MPK4–WRKYs regulatory module (Figure 3B). The contents of endogenous SA in three independent ZMP lines were then determined. Although there was no significant difference in the content of salicylate 2‐*O*‐β‐d‐glucoside (SAG) between ZMP and NT plants in the absence of the SMV inoculation, the free SA contents in ZMP1, ZMP3 and ZMP7 plants all exceeded 4000 ng/g FW and were significantly higher than the content in NT plants (Figure 5A). The expression of *GmMEKK2* was induced by exogenous SA in NT plants, whereas there were no significant effects in response to ethylene (ETH) and abscisic acid (ABA) treatments (Figure 5B). The RT‐qPCR showed that the expression levels of *PR1‐6*, *PR1‐7*, *TGA23* and *ICS1* varied among different ZMP lines. These SA‐responsive genes were up‐regulated in ZMP lines compared with NT plants, but down‐regulated in silenced plants to a certain extent (Figure 5C–G). The expressions of *WRKY5* and *WRKY39* were significantly down‐regulated in ZMP lines (Figure 5G,H).

### 2.6 *GmMEKK2* Triggered Reactive Oxygen Species Biosynthesis and Homeostasis

**FIGURE 6:**
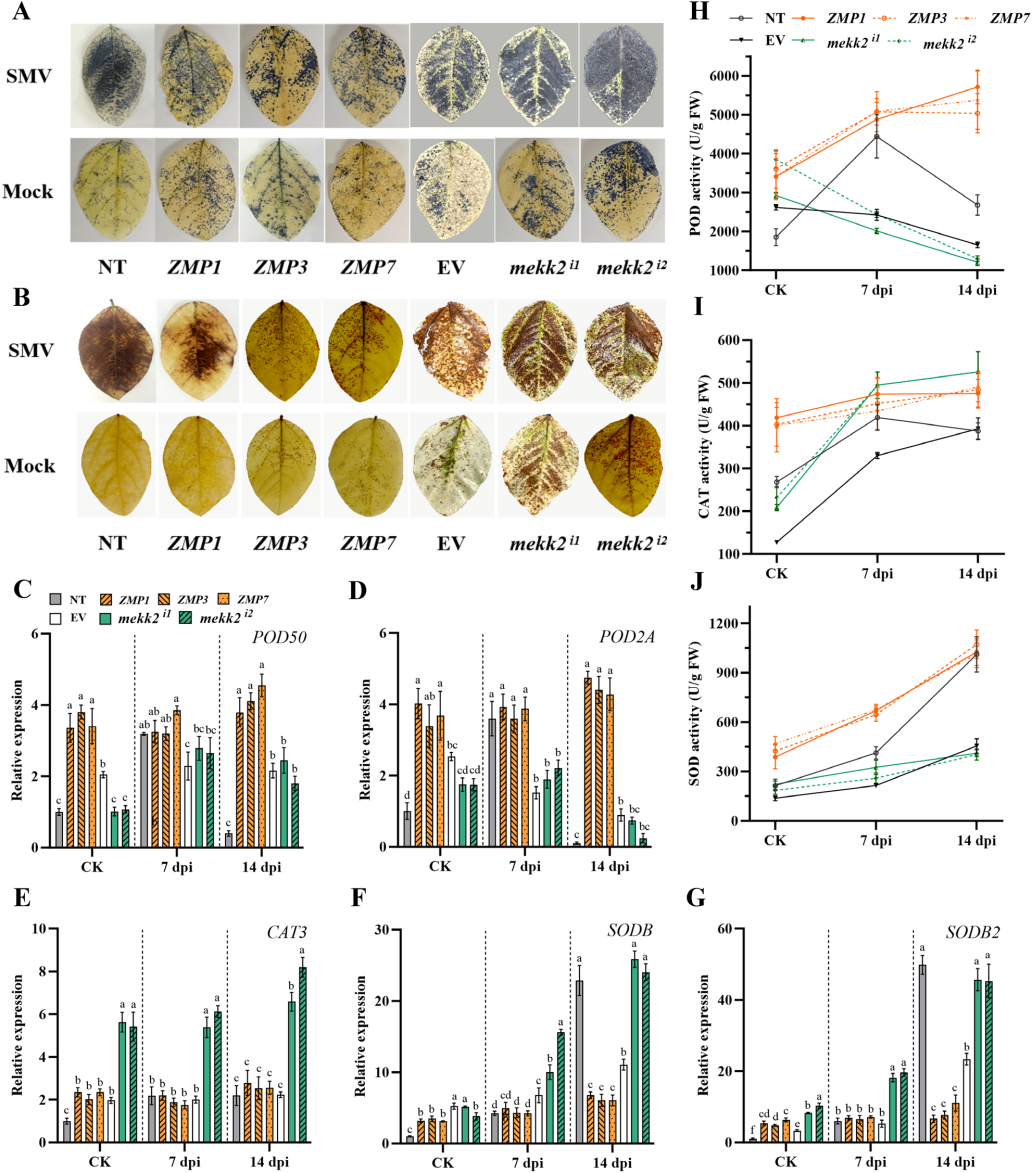
*GmMEKK2* is involved in the regulation of reactive oxygen species homeostasis in soybean. (A, B) H_2_O_2_ and O^2−^ levels in leaves were detected at 7 days post‐inoculation (dpi) using 3,3′‐diaminobenzidine (DAB) and nitroblue tetrazolium (NBT) staining, respectively. The mock‐inoculated leaves were sampled as controls. (C–G) Trends in the gene expression of antioxidases were measured after soybean mosaic virus (SMV) infection. CK, noninoculated control (H–J) Antioxidase activities were measured. POD, peroxidase; CAT, catalase; SOD, superoxide dismutase. The statistical analysis was independently performed for *GmMEKK2*‐overexpression lines ZMP1, ZMP3 and ZMP7, and gene‐silenced lines *mekk2* ^*i1*^, *mekk2* ^*i2*^ and nontransgenic (NT) plants at each stage. Values labelled with different lowercase letters are significantly different at *p* < 0.05 as determined by Duncan's test.

Plants can rapidly produce reactive oxygen species (ROS) after a viral infection through multiple pathways, such as NADPH oxidase, mitochondrial and other peroxidases, to transmit stress signals or eliminate pathogens (Torres and Dangl 2005). The transcriptome analysis showed that *GmMEKK2* and SMV inoculation activated the expression of genes that promote ROS production, such as *Rboh*s and *RPM1*, in the plant–pathogen interaction pathway (Figure 3B). The expression levels of *Rboh*s (100805517, 100801077 and 100775988) in *GmMEKK2*‐overexpression lines were 3.1–4.6‐fold higher than in NT plants before SMV infection. SMV inoculation also led to a significant up‐regulation of *Rboh* expression levels in NT plants, whereas they were only slightly elevated in ZMP plants at 14 dpi. Furthermore, the expression levels of two *OXI1* genes (100785773 and 100802487), as well as the *MPK3/6* genes (547530 and 100801339), were inhibited in ZMP plants (Figure 3B). In contrast, the expression of *MPK3/6*, which indirectly promotes ROS bursts, was significantly up‐regulated in NT plants at the early stage of the SMV infection (Figure 3B). *Rboh*s (100805517and 100775988) involved in Ca^2+^ signalling were identified as candidate genes that participate in the regulation of SMV resistance. Nitroblue tetrazolium (NBT) and 3,3'‐diaminobenzidine (DAB) staining analyses showed that there was little ROS accumulation in NT plants without SMV inoculation, but a large amount of ROS was produced, which resulted in severe oxidative damage, during the early stage of SMV infection. The accumulation of ROS in *GmMEKK2‐*silenced plants were more severe. In contrast, certain levels of H_2_O_2_ and O^2−^ accumulated in the leaves of ZMP plants when they were not infected, but the ROS content was much less than in NT plants after SMV inoculation (Figure 6A,B).

The expression levels of genes that maintain cellular ROS homeostasis, such as *POD50* and *POD2A*, were significantly higher in *GmMEKK2*‐overexpression plants than in NT and silenced plants before SMV inoculation. However, there was no significant change after SMV inoculation. However, the expression of these genes showed the tendency to increase and then decrease in NT plants (Figure 6C,D). The expression of *CAT3* in ZMP lines was slightly higher than in the NT plants, but it significantly increased in the silenced plants (Figure 6E). *SODB* and *SODB2* in NT and silenced plants were continuously expressed. The expression levels of these two genes in ZMP lines were higher than in NT plants before infection, but the levels increased slowly after infection. However, they were still significantly lower than in the NT at 14 dpi (Figure 6F,G). The peroxidase (POD) and catalase (CAT) activities in NT plants were also significantly lower than those of ZMP plants, with a significant increase only at 7 dpi (Figure 6H). *mekk2* ^*i1*^ and *mekk2* ^*i2*^ exhibited continuously declining POD activities and steadily increasing CAT activities (Figure 6H,I). The superoxide dismutase (SOD) activity in ZMP plants increased along with the SMV infection and was significantly higher than that of NT plants before SMV inoculation and at 7 dpi. Silenced plants maintained lower SOD activity levels (Figure 6J). Thus, the overexpression of *GmMEKK2* makes plants obtain higher background ROS levels, thereby enhancing resistance to SMV. On the other hand, *GmMEKK2* also efficiently maintained ROS homeostasis through the *MAPK* cascade and antioxidant enzyme system after SMV inoculation.

## 3 Discussion

In order to cope with and prevent pathogen infection, plants have evolved multilayered immune systems, including pathogen‐associated molecular pattern (PAMP)‐triggered immunity (PTI) and effector‐triggered immunity (ETI). PTI is the first layer of plant immunity and is essential for plant resistance to various pathogens (DeFalco and Zipfel 2021). ETI typically triggers localised cell death at the site of infection, called the hypersensitive response, which limits pathogen proliferation (Alhoraibi et al. 2019). There is growing evidence that PTI and ETI reinforce each other to refine the plant immune system. The MAPK cascade also plays a crucial role in this PTI–ETI cooperation (Zhai et al. 2022; Jacob et al. 2023). As the family with the most members in the cascade, at least 10 MAPKKKs have been identified as components of the typical MAPK cascades involved in the regulation of plant immune responses (Wang et al. 2018). Activated MPK6 phosphorylates MAPKKK5, forming a positive feedback mechanism to further enhance plant disease resistance (Bi et al. 2018). In contrast, MEKK1 is regarded as a negative regulator of plant immunity, and mutations of *MEKK1*, as well as of any component of its downstream MKK1/2–MPK4 cascade, could enhance plant disease resistance (Bi et al. 2018).

Here, we determined GmMEKK2 to be evolutionarily conserved with other identified MAPKKKs, sharing 76% homology with the *Arabidopsis* homologous protein MEKK2. It was classified into the MEKK subfamily due to its G (T/S)Px(W/Y/F)MAPExV conserved motif (Figure S1), which is different from the GTXX (W/Y) MAPE motif of Raf and GTPEFMAPE (L/V) Y of ZIK (Yin et al. 2013; Wang et al. 2016). Four independent single‐copy *GmMEKK2*‐overexpression lines, ZMP1, ZMP3, ZMP6 and ZMP7, were obtained. The expression of *GmMEKK2* among these four transgenic lines was higher than that of the NT line (Figure S2E). The expression levels of exogenous gene promoters are affected by the positions or co‐suppression effects of homologous genes (Napoli et al. 1990; Shan et al. 2014), causing the inhibition of the expression of transformed exogenous genes. In this study, the *GmMEKK2* gene had homologous nucleic acid sequences in soybean, which led to DNA–DNA and/or DNA–RNA interactions. In addition, multiple copies were detected in lines ZMP2, ZMP4 and ZMP5 by Southern blotting (Figure S2F), which may explain the decreased expression levels in ZMP4 and ZMP5. The overexpression of *GmMEKK2* not only reduced the SMV DI but also decreased the SMV quantity in soybean (Figure 2B,C). Moreover, *GmMEKK2* overexpression increased yield‐related traits in soybean (Figure 2E). In contrast, the disease susceptibility of silenced plants constructed through VIGS was significantly more severe than that of the EV‐treated control (Figure 1). These results are consistent with the findings of Sun et al. (2014) on *BnaMAPKKK19*. *BnaMAPKKK19* may mediate cell death through *BnaMKK9* in response to mycorrhizal stimulation in oilseed rape (Sun et al. 2014). Additionally, *MAPKKK5* enhances *Arabidopsis* resistance to powdery mildew and *Pseudomonas syringae* (Fernandez‐Milmanda 2023; Wang, Chen, et al. 2023) by phosphorylating the MKK4/5–MPK3/6 cascade.

Transcriptionally, *GmMEKK2*‐overexpressing soybean was virtually unaffected by SMV inoculation. Similar to the research of Hawk et al. (2024), the transcriptional profile of wild‐type roots exhibits significantly more pronounced changes post‐infection than those of *KD‐GmMKK2*‐overexpression samples. Pathogen proliferation and effectors can influence the plant carbon metabolism capacity and interfere with nitrogen transport or assimilation in the plants (Cai et al. 2023). In our study, the expression of DEGs involved in carbohydrate metabolism, nitrogen metabolism, and the photosynthetic system was significantly suppressed in the NT, whereas these basic metabolism‐related genes in ZMP plants showed little change (Table S5). Additionally, pathogen infection may alter the endoreplication process, disrupt the cell cycle, and lead to genomic instability (Tuteja et al. 2011; Choi et al. 2015; Shang et al. 2023). In the NT_14d samples, which exhibited the most severe symptoms, a significant up‐regulation of genes involved in DNA repair and the maintenance of genomic stability was indeed detected (Table S7). Soybean plants overexpressing *GmMEKK2* were better able to maintain normal growth and physiological functions after SMV inoculation.

MEKK2 has been identified as a suppressor of MKK1/2 that enhances disease resistance by blocking the phosphorylation of MPK4 (Bigeard et al. 2015; Nitta et al. 2020). Here, GmMEKK2 lacked phosphorylation activity but interacted with GmMKK1 and GmMPK4A, which are the components of the MEKK1–MKK1/2–MPK4 cascade (Figure 4F–I), leading to signal transduction blockage and thereby promoting the expression of GmSUMM2 (Figure 4D). SUMM2 serves as a monitor of MAPK cascade signalling. When a component is bound by pathogen proteins, causing the cascade signal to be blocked, the SUMM2 protein activates downstream immune responses (Genot et al. 2017). SUMM2 forms a positive feedback loop with CRCK3, MEKK2 and MPK4, allowing MEKK2 to further amplify immune signals by promoting *SUMM2* expression (Genot et al. 2017; Nitta et al. 2020). This is consistent with the observation that *GmSUMM2* expression was significantly enhanced in *GmMEKK2‐*overexpression plants and down‐regulated in silenced plants (Figure 4D). This also indicates that the function of *GmMEKK2* is relatively conserved between soybean and *Arabidopsis*.

The MAPK cascade forms intricate interconnected networks with hormone signalling, transcription factors and R proteins to regulate plant immune responses against pathogens (Li et al. 2023). SA is a phenolic phytohormone renowned for its pivotal role in defence responses. The silencing of the immune negative regulator *MEKK1* results in a significant accumulation of SA in soybean (Xu et al. 2018). Additionally, the constitutive expression of *MPK3* in *Arabidopsis* leads to an autoimmune phenotype characterised by dwarfism, elevated SA levels and up‐regulated expression of *SUMM1/2* (Genot et al. 2017; Lang et al. 2017). Recent studies by Hawk et al. (2024) have demonstrated that the enhanced soybean cyst nematode resistance of *KD‐GmMKK2*‐overexpression plants is attributed to the activation of defence genes involved in the response to chitin, respiratory burst, and the SA‐mediated signalling pathway. The current study revealed that the overexpression of *GmMEKK2* promoted endogenous SA synthesis (Figure 5A) and activated the SA‐induced defence response (Figure 3B). SA signalling is mediated by the transcriptional regulator NPR1, which can shuttle from the cytoplasm to the nucleus in a redox‐dependent manner and is dependent on thioredoxins (Zhang et al. 2003; Tada et al. 2008). Nuclear NPR1 then associates with TGA transcription factors to induce the expression of SA‐dependent genes (Mou et al. 2003). *NPR1*, *TGA* and SA‐responsive genes, such as *PR1‐6* and *PR1‐7*, were significantly up‐regulated in *GmMEKK2‐*overexpression plants (Figure 5C–E). Two genes encoding WRKY5 and WRKY39 were identified as negative regulators of SA‐mediated defence responses and can be used for the gene editing‐based breeding of resistant varieties.

ROS are molecules that conduct stress signals and also have a role in scavenging pathogens. They are either byproducts of photosynthesis or induced during abiotic stress and pathogen intrusion (Apel and Hirt 2004). In the H_2_O_2_ regulatory pathway, MPK3 is activated by OXI1, which emerged as having potential roles in hypersensitivity and disease resistance (Rentel et al. 2004). OXI1 contributes significantly to ROS accumulation (Rentel et al. 2004). In the resistant cultivar L29, MPK3 exhibits extreme resistance to SMV‐G5H mediated by Rsv3 (Alazem et al. 2023). In our study, we found that the expression of two *OXI1* genes, as well as *MPK3/6* genes, were significantly suppressed in soybean overexpressing *GmMEKK2* (Figure 3B). Meanwhile, genes involved in the Ca^2+^ signalling pathway, such as *CNGCs* and *Rbohs*, which participate in plant defence responses, were weakly activated in ZMP plants (Figure 3B). This accounts for the higher basal ROS level in ZMP plants compared to NT plants (Figure 6A,B). *MPK3/6* was significantly up‐regulated in the NT plants during the early stages of SMV infection, which triggered a burst of ROS (Figure 3B) and led to extensive cell death. Recent studies showed that CNGCs (Cyclic Nucleotide‐Gated Channels) control the accelerated influx of Ca^2+^ into the plant cell in the presence of PAMPs (Sun et al. 2021). There is also substantial evidence indicating that *Rbohs* enhance plant resistance to biotic stress (Nozaki et al. 2013; Otulak‐Kozieł et al. 2020; Marcec and Tanaka 2021). In addition, the expression levels of *POD50*, *POD2A*, *CAT1* and *CAT3* genes, which are essential for the maintenance of ROS homeostasis, were up‐regulated in ZMP plants (Figure 6C–G). Correspondingly, the activities of POD, CAT and SOD enzymes in ZMP plants were higher than in the NT (Figure 6H). Therefore, plants overexpressing *GmMEKK2* contained relatively high levels of ROS to enhance immunity to SMV. Conversely, it efficiently maintained ROS homeostasis through the *MAPK* cascade and antioxidase system to improve resistance after SMV inoculation.

**FIGURE 7:**
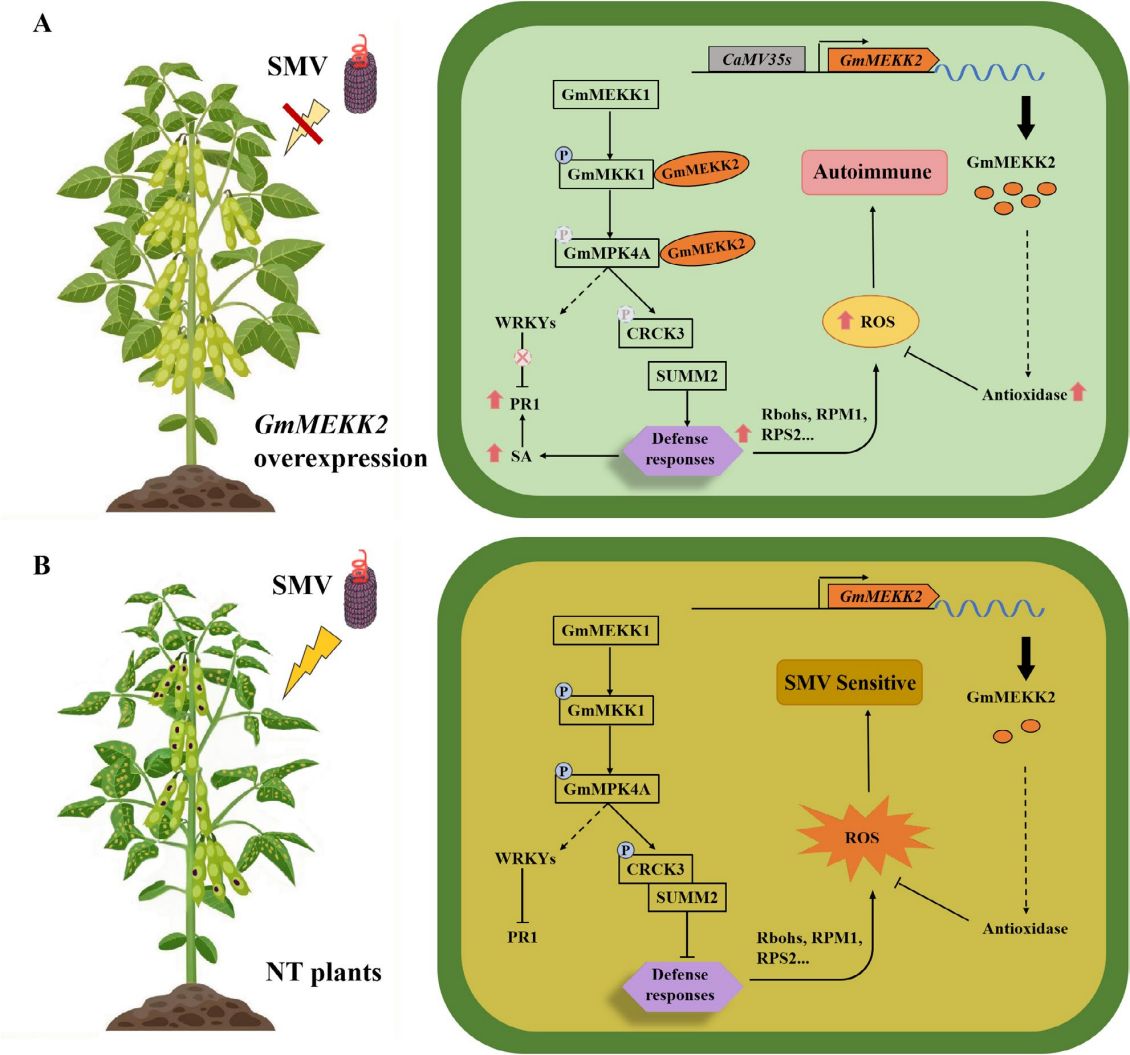
Molecular mechanisms underlying the GmMEKK2‐mediated regulation of soybean mosaic virus (SMV) resistance in soybean. (A) Phenotype and regulatory mechanism of *GmMEKK2*‐overexpression plants under SMV inoculation. Left: *GmMEKK2*‐overexpression plants (ZMP) show no visible SMV symptoms with autoimmunity phenotype such as leaf yellowing. Right: In ZMP plants, GmMEKK2 (orange ellipses) interacts with GmMKK1 and GmMPK4A, blocking the phosphorylation (letter P in a blue circle) of the GmMEKK1‐GmMKK1‐GmMPK4A cascade. This inhibition represses (cross in a red circle) WRKY transcription factors and leads to non‐phosphorylated CRCK3 releasing SUMM2. This then triggers defence responses such as salicylic acid (SA)‐induced gene expression and basal reactive oxygen species (ROS) accumulation. The elevated ROS constitutivly results in autoimmunity in ZMP plants. (B) Left: Nontransgenic (NT) plants exhibit severe SMV symptoms such as mosaic leaves and mottled pods. Right: In NT plants, *GmMEKK2* expression is low, so the GmMEKK1‐GmMKK1‐GmMPK4A cascade remains active. GmMPK4A phosphorylates CRCK3, which binds with and represses SUMM2. This suppresses defence responses, and leads to a ROS burst.

In summary, GmMEKK2 does not possess typical kinase functions. However, it can activate and amplify SUMM2‐mediated ETI by blocking the MEKK1–MKK1/2–MPK4 cascade signalling in soybean. In overexpression plants, GmMKK1 and GmMPK4A were bound with GmMEKK2 and phosphorylation was inhibited, leading to the activation of SUMM2‐related downstream defence responses, such as the up‐regulated expression of the SA‐induced defence gene *PR1* and ROS accumulation, ultimately resulting in autoimmunity (Figure 7A). This autoimmunity partially prevents SMV infection. In contrast, NT plants initiated immune responses after SMV infection, and this delayed response allowed the virus to replicate within the plants, causing varying degrees of symptoms, such as mosaicism and necrosis (Figure 7B). Compared to previous varieties with single resistance genes (Ma et al. 2016; Alazem et al. 2023), soybean overexpressing *GmMEKK2* possessing autonomous immune responses exhibited varying degrees of resistance to several SMV strains such as SC3, SC5, SC7 and SC8 (Figure S4). Additionally, overexpression of this gene did not affect agronomic traits such as yield, pod number and 100‐seed weight (Figure 2E). Our research expands the adaptability range, providing critical theoretical support for further breaking the bottleneck of SMV resistance breeding and enhancing the competitiveness of the soybean industry.

## 4 Experimental Procedures

### 4.1 Plant Materials and Growth Conditions

Soybean cultivar Tainlong 1, bred at the Institute of Oil Crops, Chinese Academy of Agricultural Sciences, Wuhan, China, was used as a transformation receptor, and as a nontransgenic control for all experiments in this study. The transgenic plants and nontransgenic Tianlong 1 (NT) were all pot‐cultivated in a walk‐in greenhouse at the Zijingang campus experimental farm of Zhejiang University, Hangzhou, China. The plants were grown at 28°C/20°C ± 1°C (day/night) with a 16 h photoperiod under fluorescent white light. The plants were supplied daily with 200 mL half‐strength Hoagland's nutrient solution.

### 4.2 *GmMEKK2* Isolation and Phylogenetic Analysis

The full‐length *GmMEKK2* cDNA was amplified by PCR using the gene‐specific primer pair *GMEKK_CD* (Table S1). The phylogenetic analysis of *GmMEKK2* was performed using MEGA v. 5.0 with neighbour‐joining techniques. The *MAPKKK* homologous gene sequences from *Glycine soja* and other related species were obtained from the NCBI database (https://www.ncbi.nlm.nih.gov). The neighbour‐joining tree was constructed based on the full‐length MAPKKK protein sequences to investigate their evolutionary relationships using MEGA v. 5.0 (Tamura et al. 2011). The bootstrap test method was adopted, and the replicate was set to 1000. The multiple sequence alignment was generated using ClustalX software (Thompson et al. 2002).

### 4.3 Plant Transformation and Transgenic Plant Identification

The *GmMEKK2* full‐length coding DNA sequence (CDS) was inserted into pDONOR221 (Invitrogen) and then transferred to a pB7FWG2 vector via an LR recombination reaction in the Gateway system. The reconstructed pB7FWG2 vector contained the *GmMEKK2* target gene and the *bar* gene, which encodes phosphinothricin acetyltransferase and acts as a selection marker. The constructed vector was transformed into *A. tumefaciens* EHA101, which was subsequently used to transform the soybean cultivar Tainlong 1 through an optimised *A. tumefaciens* ‐mediated transformation system (Yang et al. 2016). The putative transgenic T_0_ plants were identified, and the positive transgenic plants were self‐pollinated to generate T_1_ and T_2_ progeny.

Basta painting, LibertyLink strip analysis, and PCR were used to identify the putative transgenic soybean plants. For Basta painting, the fully enlarged trifoliate leaves of putative transgenic and NT plants were painted with 135 mg/L Basta solution for 7 days. A LibertyLink strip was used to determine the phosphinothricin acteyltransferase protein levels in putative transgenic plants in accordance with the manufacturer's instructions (Envirologix Inc.). Genomic DNAs from putative transgenic and NT leaves were extracted using a simple homogenisation and ethanol precipitation method. Target sequences were amplified via PCR with the specific primer pairs for *bar* and for the *GMEKK_TDNA* that included the T‐DNA fragment containing *GmMEKK2*. The DNAs of the pB7FWG2‐*GmMEKK2* plasmid and NT plants were used as the positive and negative controls, respectively.

### 4.4 Southern Blot Analysis

A Southern blot analysis was conducted following the protocol Southern (2006) with minor modifications using digoxigenin (DIG)‐labelled *GmMEKK2*‐specific and *bar*‐specific probes. The genomic DNAs were extracted from T_0_ *GmMEKK2* transgenic and NT leaves using the CTAB method (Allen et al. 2006). The extracted DNAs were each digested with the restriction enzyme XbaI. Southern blots were hybridised with a DIG‐labelled *GmMEKK2*‐ and *bar*‐specific probe at 68°C for 16 h, followed by detection as per the protocol of the High Prime DNA Labeling and Detection Starter Kit I (Roche).

### 4.5 RT‐qPCR

Gene relative expression levels were assessed by RT‐qPCR analyses. The top fully expanded trifoliolate leaves were sampled, placed in liquid nitrogen, and stored at −80°C for RNA extraction. Total RNA was extracted using TRIzol reagent (TaKaRa). The first‐strand cDNA was synthesised by reverse transcription using HiScriptTMQ RT SuperMix for qPCR (Vazyme). The qPCR analyses were performed based on the SYBR Premix Ex Taq II (Vazyme) manufacturer's protocol with specific primers. The soybean *Actin11* gene served as a control, and the mRNA levels for each cDNA probe were normalised to the *Actin11* mRNA (Xue et al. 2012). The qPCR analyses were conducted on a CFX96 Real Time System (Bio‐Rad) machine, and the gene relative expression levels were calculated from three technical and biological replicates per sample using the 2^−ΔΔ*C*t^ method (Livak and Schmittgen 2001). All the primers used in the RT‐qPCR analyses are listed in Table S1.

### 4.6 SMV Inoculation and Disease Index DIAssays

The predominant SMV strains that are prevalent in China, SC3, SC5, SC7 and SC8, were used for rub‐inoculation. Briefly, 14‐day‐old soybean plants were inoculated with SMV as follows: each virus‐infected soybean leaf was mixed with carborundum and ground into a homogenate using 0.01 M phosphate buffer at pH 7.3 in an ice bath. Then, 1 mL of the mixture was placed on a small paint brush and used to rub the first trifoliate leaf. The leaf was then washed with buffer after 1 h. The control group was mock‐inoculated with buffer. All the plants were isolated using anti‐aphid mesh. Nine NT and nine transgenic plants were inoculated, with three repeats. The top trifoliate leaves were sampled at 0, 3, 7, 14 and 21 dpi to detect the relative expression of *GmMEKK2* induced by SMV infection in NT plants as assessed by RT‐qPCR with primer pair *GMEKK2_qRT*. The viral contents in NT and transgenic plants at 7, 14 and 21 dpi were determined, and the SMV‐sensitive cultivar 1138‐2 was sampled as a positive control. RT‐qPCR with primer pair *CP_SC* was performed to detect the relative expression level of the *CP* gene in SMV to represent the relative viral content. Three biological replicates were performed for each sample.

The SMV disease index (DI) was calculated at 21 dpi according to the method described in Zhi and Gai (2004). The SMV symptoms of each soybean plant were divided into five levels from 0 to 4 and the DI = [∑(number of plants at each level × corresponding level)/total number of plants investigated × the highest level] × 100. A total of 21 plants per line were used for the DI calculations. In addition, yield traits 100‐seed weight, number of pods per plant and yield per plant were determined after harvest.

### 4.7 DAS‐ELISA Assay

The top fully expanded trifoliate leaves from soybean at 21 dpi were subjected to a DAS‐ELISA (AC Diagnostics Inc.). It was performed in accordance with the manufacturer's instructions. The A_405nm_ of SMV‐infected samples and mock‐inoculated plants were measured. If the ratio of the sample to the negative control was higher than 2, then the sample was regarded as susceptible; otherwise, it was considered resistant. Three replicates, totaling 15 SMV‐infected and 15 mock‐inoculated plants, were tested.

### 4.8 Transcriptome Sequencing and Data Processing

The NT and the *GmMEKK2‐*overexpression line ZMP3 were selected for RNA‐sequencing based on the results of the SMV resistance assay. The top trifoliate leaves at 7 and 14 dpi with SMV strain SC7 were sampled, and the leaves before inoculation were used as controls. The six tissues were labelled as NT_CK, NT_7d, NT_14d, ZMP_CK, ZMP_7d and ZMP_14d. Three replicates were sampled for each tissue. We sent 1 μg RNA per sample for library preparation using the NEBNext RNA Library Prep Kit (Illumina). The library was sequenced on an Illumina HiSeq2000 instrument. Approximately 40.7–46.8 million 150‐bp paired‐end reads were generated for each sample. After filtering low‐quality data, clean reads were retained for subsequent analysis, with the average Q30 (percentage of bases with Phred score ≥ 30) of all samples exceeding 90%.

The clean reads were obtained and then mapped to the soybean reference genome (Wm82a2.v1) using HISAT2 v. 2.0.5 (Kim et al. 2019). Fragments per kilobase per million was calculated for estimating the gene expression level using Stringtie v. 1.3.3 and Feature Counts v. 1.5.0 (Liao et al. 2014). A principal component analysis was performed using the DESeq2 R package (Love et al. 2014). DEGs were filtered with log_2_(fold change) > |1| and adjusted *p*‐value (*p*_adj_) < 0.05. A gene ontology (GO) enrichment analysis and a KEGG pathway enrichment analysis of DEGs were performed using Cluster profiler R package (Yu et al. 2012; Langfelder and Horvath 2008; Doncheva et al. 2019).

### 4.9 In Vitro Phosphorylation Assay

To test the phosphorylation of GmMEKK2, recombinant proteins GST‐GmMEKK2, GST‐GmMEKK2^K36E^, GmMEKK1‐FLAG (positive control) and GmMEKK1^K321M^‐FLAG (negative control) were expressed and purified from *E. coli* BL21. The in vitro phosphorylation assay was performed as described previously with slight modifications (Peng et al. 2020). Briefly, 1 μg protein was incubated with 10 μM ATP in 30 μL reaction buffer (50 mM Tris–HCl pH 7.5, 5 mM MgCl_2_ and 1 mM dithiothreitol [DTT]) at 30°C for 30 min. Reactions were terminated with 5× SDS loading buffer. The autophosphorylation was analysed by α‐pSer/Thr antibody (ABclonal), and total protein levels were assessed by Coomassie brilliant blue staining.

### 4.10 Y2H Assay

To perform the Y2H assay, the CDSs of *GmMEKK2*, *GmMKK1* (GeneID: 547701), *GmMPK4A* (GeneID: 100779469) and *GmMPK13‐like* (GeneID: 100786314) were amplified by PCR and then independently cloned into the pGADT7 or pGBKT7 vectors. Constructs GmMEKK2‐AD, GmMKK1‐BD, GmMPK13‐like‐BD and GmMPK4A‐BD were each transformed into the competent yeast cells, which were then grown on selection media SD/−Leu/−Trp and SD/−Leu/−Trp/−His/−Ade to confirm the interactions (Bao et al. 2024). The primers used are listed in Table S1.

### 4.11 GST Pull‐Down Assay

For pull‐down assays, the *GmMEKK2* CDS was cloned into the pGEX6P‐1 vector digested with EcoRI and XhoI, whereas GmMKK1, GmMPK4A and GmMPK13‐like CDSs were each cloned into pET‐28a digested with EcoRI and SalI. Recombinant proteins GST‐*GmMEKK2*, GmMKK1‐His, GmMPK4A‐His and GmMPK13‐like‐His were each expressed in *E. coli* BL21 by induction with 0.5 mM IPTG at 16°C for 16 h, followed by purification using MagBeads (GeneCreate). Assays were performed as described in Yin et al. (2023), with protein interactions detected by western blotting using anti‐His and anti‐GST (ABclonal) antibodies.

### 4.12 Silencing of *GmMEKK2*

*GmMEKK2* gene expression was knocked down in soybean using TRV‐based VIGS (Liu et al. 2020). Two fragments of 166 and 165 bp encoding the kinase domain sequence were selected and independently cloned into the pTRV2 vector. Then, 7‐day‐old seedling leaves were infected with *Agrobacterium* containing either pTRV1/pTRV2‐*GmMEKK* ^i1^ (*mekk2* ^*i1*^ treatment) or pTRV1/pTRV2‐*GmMEKK* ^i2^(*mekk2* ^*i2*^ treatment). The empty vector (EV) was used as the control. Each treatment included 15 pots, with three plants per pot. The primers used are listed in Table S1.

At 7 days post‐silencing treatment, plants were inoculated with SMV strain SC7. The relative viral contents and *GmMEKK2* expression levels were measured at 0, 7, 14 and 21 dpi. The DI was analysed at 21 dpi.

### 4.13 Exogenous Hormone Treatments and Endogenous SA Detection

The 14‐day‐old NT soybean seedlings were subjected to exogenous hormone spraying. The seedlings were treated with 1 mM SA, 100 μM ABA and 200 μM ETH, individually. The fully expanded apical leaves were harvested to extract RNA at 0, 3 and 6 h after treatment. The relative expression of *GmMEKK2* was determined using RT‐qPCR with the specific primer pair *GMEKK2_qRT*. Three biological replicates were performed for each treatment.

Endogenous SA was extracted from the top trifoliate leaves of *GmMEKK2*‐overexpression lines and NT plants. The content was detected using a Waters1525 high‐performance liquid chromatograph with a fluorescence detector under an excitation wavelength of 294 nm and an emission wavelength of 426 nm (Verberne et al. 2002). The bound form of SA, SA‐2‐*O*‐β‐D‐glucoside (SAG), was quantified by conversion into free SA through acid hydrolysis.

### 4.14 DAB and NBT Staining and Antioxidase Activity Assay

The H_2_O_2_ and O^2−^ contents in leaves were qualitatively detected at 7 dpi by DAB and NBT staining, respectively (Kumar et al. 2013). H_2_O_2_ production was visualised as a reddish‐brown precipitate in cleared leaves, whereas O^2−^ production was visualised as a dark‐blue precipitate.

The enzymatic activities of SOD, CAT, and POD were measured by NBT illumination, guaiacol reaction and oxidation–reduction methods, respectively (Wang et al. 2021). The expression of related genes was quantified via RT‐qPCR. The leaves from NT and transgenic plants were sampled at each stage, and three replicates were performed.

### 4.15 Statistical Analysis

SPSS Statistics 20 (IBM) and Excel (Microsoft) were used for data collation and statistical analysis in this study. The statistical analysis of different samples was performed by one‐way ANOVA with Duncan's test. A value of *p* < 0.05 was used to indicate statistical significance.

## Author Contributions

**Xuanbo Zhong:** conceptualization; formal analysis; investigation; writing – original draft preparation. **Guixiang Tang:** conceptualization; resources; funding acquisition. **Jingxiang Luo:** investigation; writing – review and editing. **Longlong Hu:** investigation; visualisation. **Yue Shu:** writing – review and editing. **Yucheng Ruan:** writing – review and editing. All authors have read and approved the current version of the manuscript.

## Funding

This work was supported by the Key Research Foundation of the Science and Technology Department of Zhejiang Province, 2021C02064‐5‐5. Zhejiang Key Laboratory of Crop Germplasm Innovation and Utilization Open Fund.

## Conflicts of Interest

The authors declare no conflicts of interest.

## Supporting information


**Figure S1:** Phylogenetic analysis and sequence alignment of MAPKKKs. (A) Phylogenetic analysis of GmMEKK2 and its homologues. The unrooted tree was constructed using the MEGA7.0 program with the neighbour‐joining (NJ) method and 1000 bootstrap replicates. The numbers at nodes represent the percentage of bootstrap scores, and the scale bar signifies 0.05 estimated amino acid substitutions per site. GmMEKK2 is highlighted with a red box. (B) Sequence alignment of MAPKKKs homologues performed by Clustalx. The conserved signature motif is highlighted with a red box. The symbols ‘*’, ‘:’, ‘.’ and space represents the degree of the site conservation from high to low. Accession number of homologous gene: GmMEKK2 (LOC100798607), GmMEKK1 (LOC100819999), GmMAPKKK5‐like_(LOC100793654), AtMEKK1 (AT4G08500), AtMEKK2 (AT4G08480), AtMEKK3 (AT4G08470), AtMAPKKK5 (AT5G66850), OsMAPKKK1_(LOC4333845), OsMAPKKK5_(LOC4334214), GsMAPKKK17‐like (LOC114412411), CaMAPKKK17‐like (LOC101490231), MtMAPKKK17‐like (LOC25480496).

**Figure S2:** The identification of transgenic soybean plants overexpressing *GmMEKK2* (*ZMPs*). (A) Schematic diagram of recombinant plasmid components containing the selection marker gene *bar* and *GmMEKK2*. LB, T‐DNA left border; RB, right border; P35S, Cauliflower mosaic virus (CaMV) 35S promoter; T35S, terminator. (B) Results of the phosphinothricin coating method. (C) Detection results of bar protein quick dip strip. (D) PCR validation of the positive transgenic lines by specific primers for *bar* gene and T‐DNA fragment containing the *GmMEKK2* gene. (E) Relative expression of the *GmMEKK2* gene in independent transgenic lines and non‐transganic plants (NT). Values labelled with different letters (a–c) are significantly different at *p* < 0.05 as determined by Duncan's test. (F) The transgene copy number determined by Southern blot analysis in T_0_ transgenic and NT plants. 1, 2, 3, 4, 5, 6 and 7 represented individual *GmMEKK2* transgenic lines. M: DL2000 marker; +: positive control (plasmid DNA); NT, non‐transgenic plants.

**Figure S3:** Flow chart of the *A. tumefaciens* mediated transformation system in soybean. (A) Soybean seeds sterilised with chlorine gas. (B) Explants co‐cultured with *A. tumefaciae* on co‐cultivation medium (CCM). (C) Clumped buds induced on shoot induction medium (IS) containing glufosinate‐ammonium as screening agent. (D) Shoot were elongated on a shoot elongation medium (SE). (E) Roots were developed on a rooting medium (RM). (F) Putative transgenic plants were acclimatisation and transplant in soil.

**Figure S4:** mpp70184‐sup‐0004‐FigureS4.tif. *GmMEKK2* overexpression enhanced the resistance to different SMV strains. (A) Disease indices of NT and each *GmMEKK2* overexpression line. (B) Quantification of SMV relative contents in soybean leaves. The disease index and virus relative content were investigated at 21 days post SMV inoculation. SC3, SC5, SC7 and SC8 were the predominant SMV strain prevalent in China. NT was used as a control. Values labelled with different letters (a–d) are significantly different at *p* < 0.05 under the same strain treatment as determined by Duncan's test.

**Figure S5:** Principal component analysis of the RNA‐Seq data. The gene expression level of all samples was analysed using PCA, represented by FPKMs (Fragments Per Kilobase of transcript per Million mapped reads).

**Figure S6:** Expression levels of nine randomly selected genes from the RNA‐Seq data. The expression patterns of genes induced by SMV in NT and *ZMP* plants were quantified using qRT‐PCR to validate the reliability of RNA‐Seq data. The qRT‐PCR results are presented as bar charts, while the FPKMs for each sample are displayed as lines graphs. The gene ID is annotated at the top of each subgraph. NT_CK, NT_7d and NT_14d represent the non‐transgenic plants before SMV infection, 7 and 14 days after SMV infection respectively. ZMP_CK, ZMP_7d and ZMP_14d GmMEKK2 over‐expression lines before SMV infection, 7 and 14 days after SMV infection respectively.

**Figure S7:** Overview of the gene expression profile during early stages after SMV infection. (A, B) Venn diagrams of differentially expressed genes (DEGs) in NT and *ZMP*, respectively. (C) Venn diagrams of DEGs at each stage between NT and *ZMP*. (D) Counts of up‐/down‐regulated DEGs in each pairwise compared group. (E) Heatmap showing the global gene expression profile in NT and *ZMP* before and after SMV infection.

**Figure S8:** Kyoto Encyclopedia of Genes and Genomes (KEGG) enrichment analysis of DEGs. The most significant 20 KEGG pathways in each comparison group were selected to draw scatter plots for display. The size of the dots represents the number of genes annotated to the KEGG pathway, and the colour from red to purple represents the significance of enrichment.

**Table S1:** Primers used in this research.

**Table S2:** mpp70184‐sup‐0010‐TableS2.xlsx. *GmMEKK2* gene transformation.

**Table S3:** Comparison of SMV resistance levels detected by DAS‐ELISA.

**Table S4:** Mapping result of samples to the reference genome.

**Table S5:** Down‐regulated genes after SMV infection in NT plants.

**Table S6:** Up‐regulated genes at 7 day past inoculation (dpi) in NT plants.

**Table S7:** Up‐regulated genes at 14 dpi in NT plants.

**Table S8:** Common DEGs among the ZMP_CK versus NT_CK, ZMP_7d versus NT_7d and ZMP_14d versus NT_14d comparisons.

## Data Availability

The data that support the findings of this study are available in the Supporting Information of this article. RNA sequencing data have been deposited in the NCBI’s GEO under accession number GSE288208 (https://www.ncbi.nlm.nih.gov/geo/query/acc.cgi?acc=GSE288208).
