# Supplementary figures and images for: GmMEKK2 Disrupts the MKK1/2–MPK4 Cascade to Amplify Immune Signalling and Confer Enhanced Resistance to Soybean Mosaic Virus
Source: Mol Plant Pathol. 2025 Nov 30;26(12):e70184. doi: 10.1111/mpp.70184 (PMC12665255; doi:10.1111/mpp.70184)

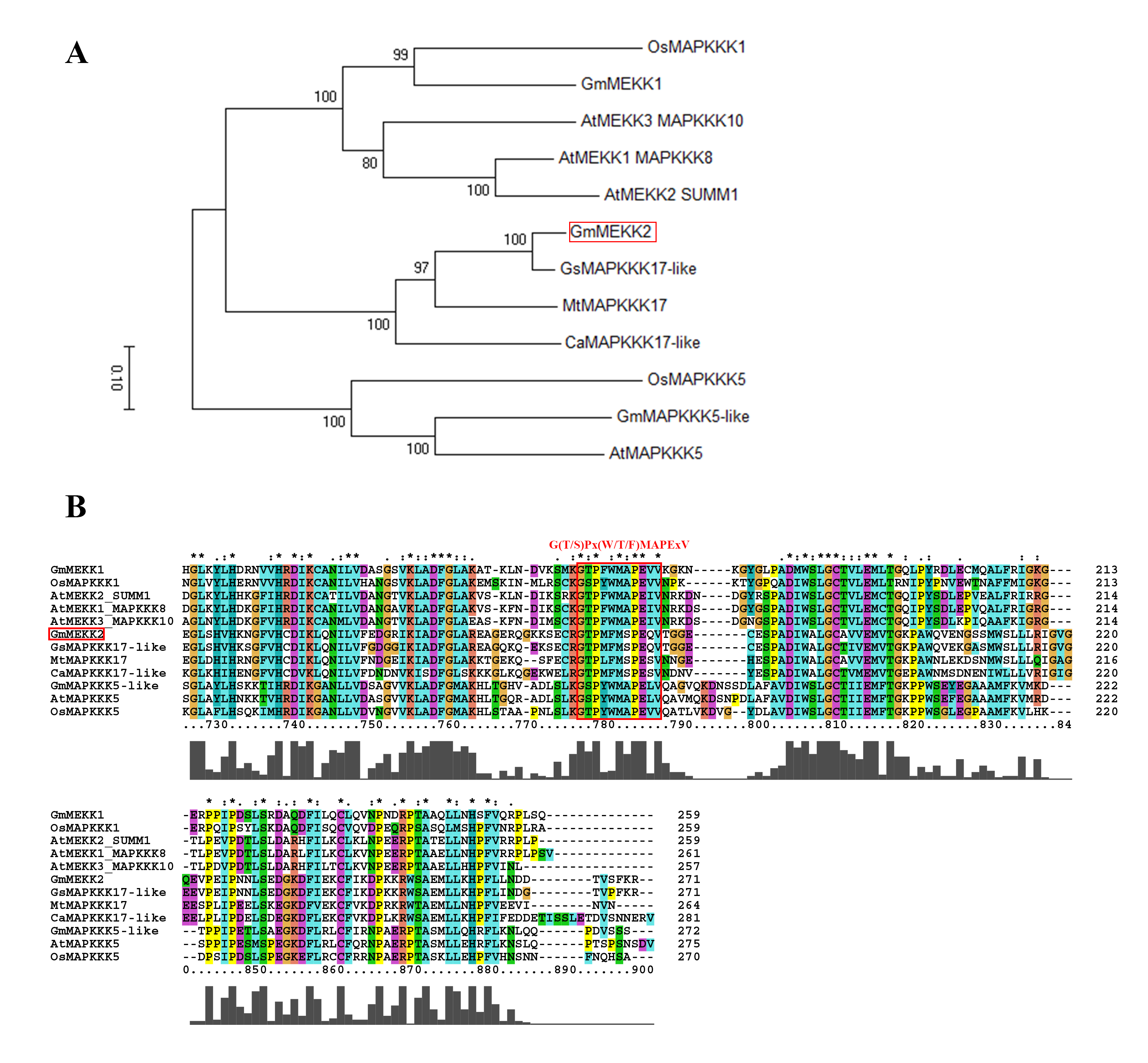

Supplement: Supplementary file 1 — Figure S1: Phylogenetic analysis and sequence alignment of MAPKKKs. (A) Phylogenetic analysis of GmMEKK2 and its homologues. The unrooted tree was constructed using the MEGA7.0 program with the neighbour‐joining (NJ) method and 1000 bootstrap replicates. The numbers at nodes represent the percentage of bootstrap scores, and the scale bar signifies 0.05 estimated amino acid substitutions per site. GmMEKK2 is highlighted with a red box. (B) Sequence alignment of MAPKKKs homologues performed by Clustalx. The conserved signature motif is highlighted with a red box. The symbols ‘*’, ‘:’, ‘.’ and space represents the degree of the site conservation from high to low. Accession number of homologous gene: GmMEKK2 (LOC100798607), GmMEKK1 (LOC100819999), GmMAPKKK5‐like_(LOC100793654), AtMEKK1 (AT4G08500), AtMEKK2 (AT4G08480), AtMEKK3 (AT4G08470), AtMAPKKK5 (AT5G66850), OsMAPKKK1_(LOC4333845), OsMAPKKK5_(LOC4334214), GsMAPKKK17‐like (LOC114412411), CaMAPKKK17‐like (LOC101490231), MtMAPKKK17‐like (LOC25480496). [file MPP-26-e70184-s002.tif]

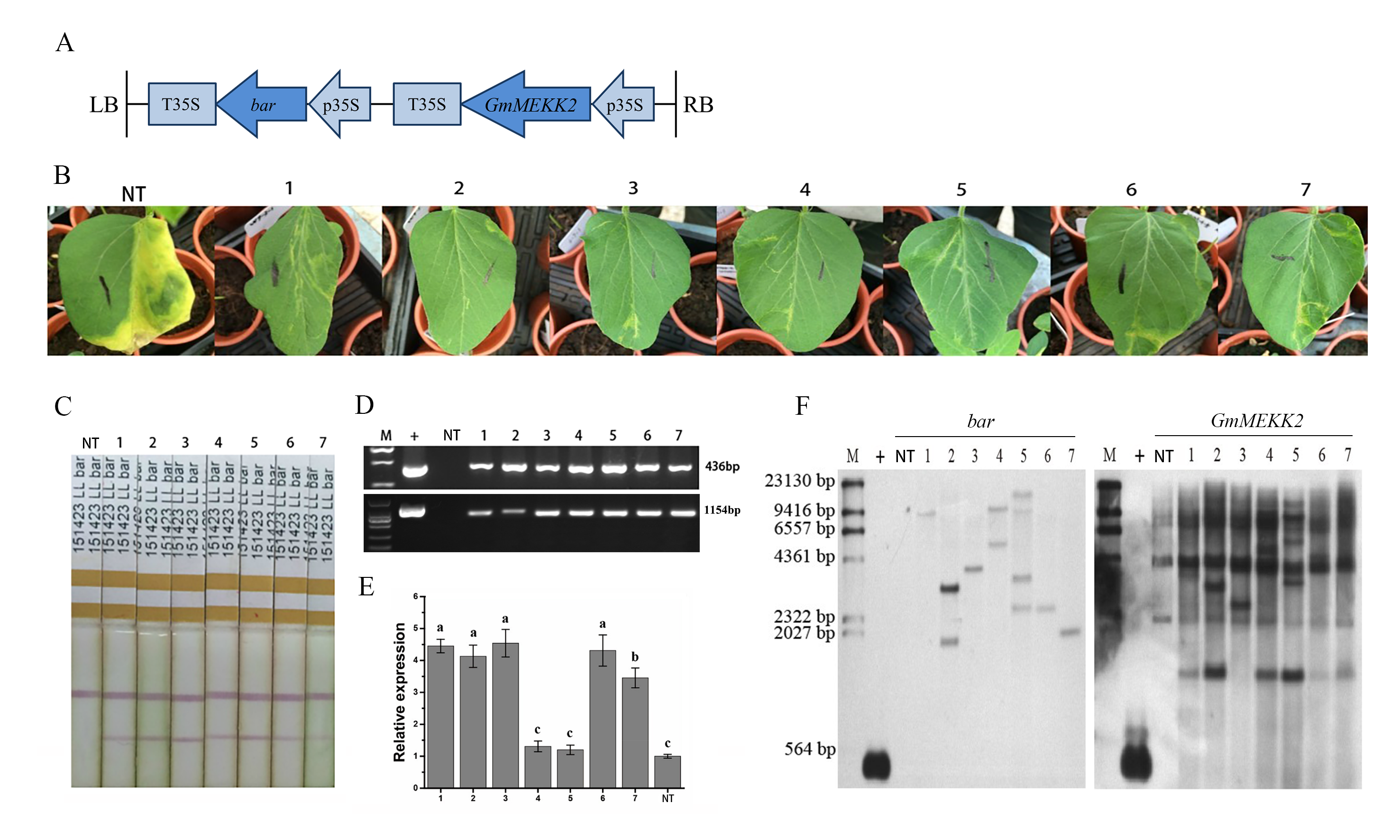

Supplement: Supplementary file 2 — Figure S2: The identification of transgenic soybean plants overexpressing GmMEKK2 (ZMPs). (A) Schematic diagram of recombinant plasmid components containing the selection marker gene bar and GmMEKK2. LB, T‐DNA left border; RB, right border; P35S, Cauliflower mosaic virus (CaMV) 35S promoter; T35S, terminator. (B) Results of the phosphinothricin coating method. (C) Detection results of bar protein quick dip strip. (D) PCR validation of the positive transgenic lines by specific primers for bar gene and T‐DNA fragment containing the GmMEKK2 gene. (E) Relative expression of the GmMEKK2 gene in independent transgenic lines and non‐transganic plants (NT). Values labelled with different letters (a–c) are significantly different at p < 0.05 as determined by Duncan's test. (F) The transgene copy number determined by Southern blot analysis in T0 transgenic and NT plants. 1, 2, 3, 4, 5, 6 and 7 represented individual GmMEKK2 transgenic lines. M: DL2000 marker; +: positive control (plasmid DNA); NT, non‐transgenic plants. [file MPP-26-e70184-s001.tif]

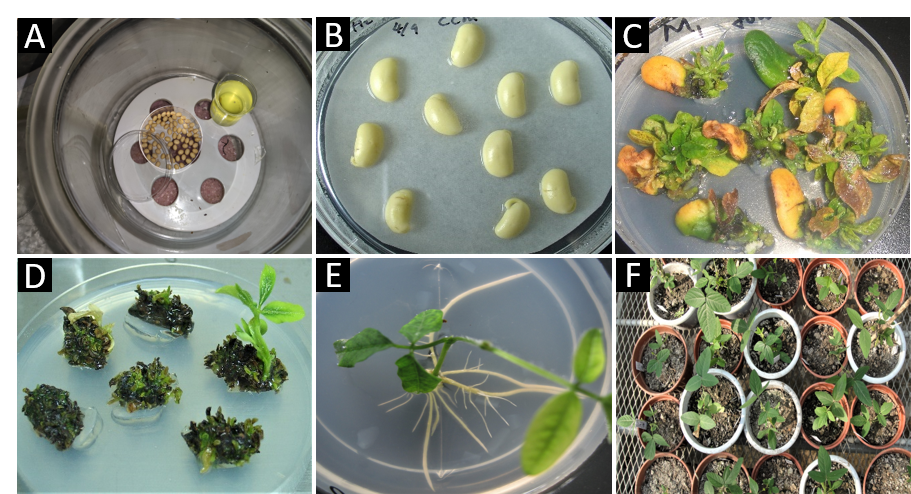

Supplement: Supplementary file 3 — Figure S3: Flow chart of the A. tumefaciens mediated transformation system in soybean. (A) Soybean seeds sterilised with chlorine gas. (B) Explants co‐cultured with A. tumefaciae on co‐cultivation medium (CCM). (C) Clumped buds induced on shoot induction medium (IS) containing glufosinate‐ammonium as screening agent. (D) Shoot were elongated on a shoot elongation medium (SE). (E) Roots were developed on a rooting medium (RM). (F) Putative transgenic plants were acclimatisation and transplant in soil. [file MPP-26-e70184-s009.tif]

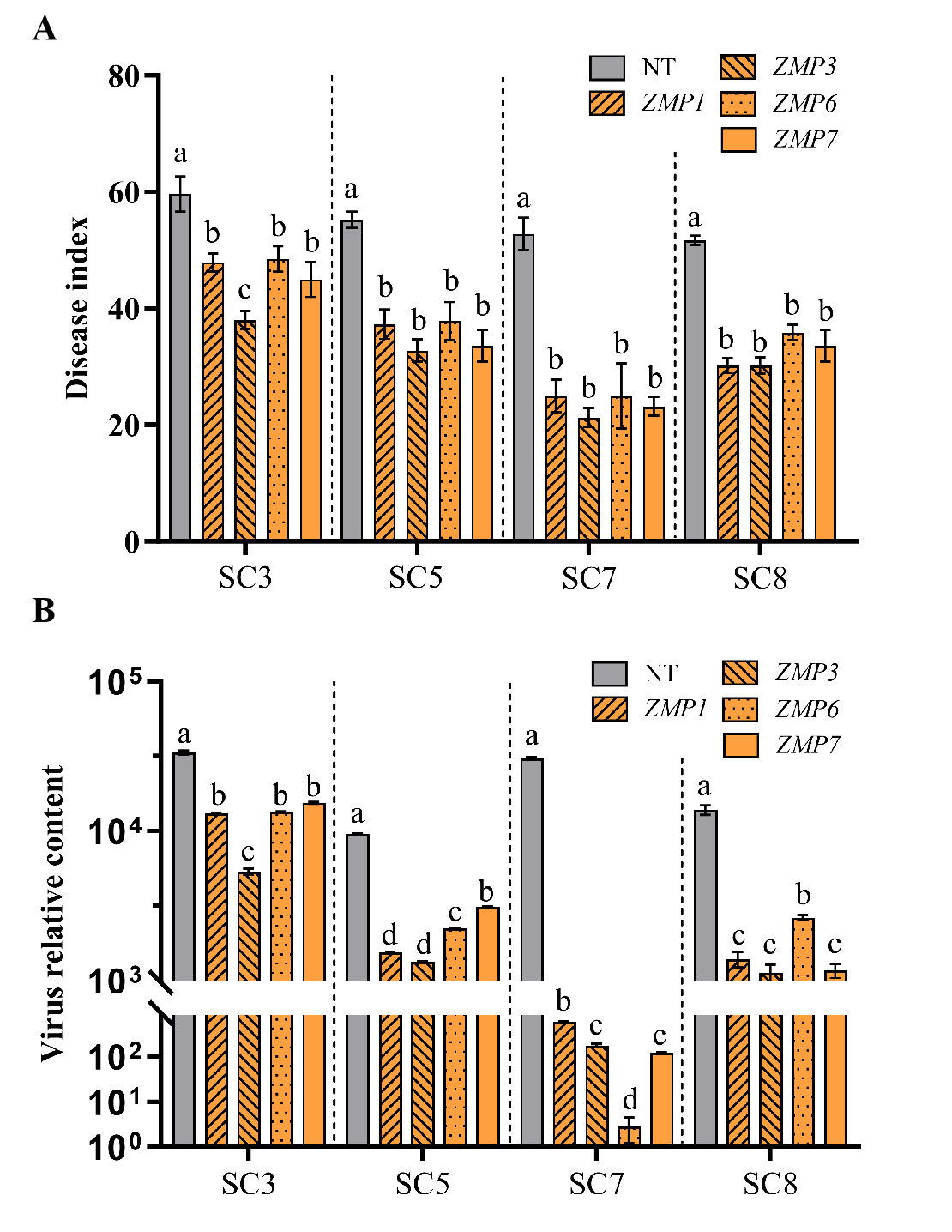

Supplement: Supplementary file 4 — Figure S4: mpp70184‐sup‐0004‐FigureS4.tif. GmMEKK2 overexpression enhanced the resistance to different SMV strains. (A) Disease indices of NT and each GmMEKK2 overexpression line. (B) Quantification of SMV relative contents in soybean leaves. The disease index and virus relative content were investigated at 21 days post SMV inoculation. SC3, SC5, SC7 and SC8 were the predominant SMV strain prevalent in China. NT was used as a control. Values labelled with different letters (a–d) are significantly different at p < 0.05 under the same strain treatment as determined by Duncan's test. [file MPP-26-e70184-s014.tif]

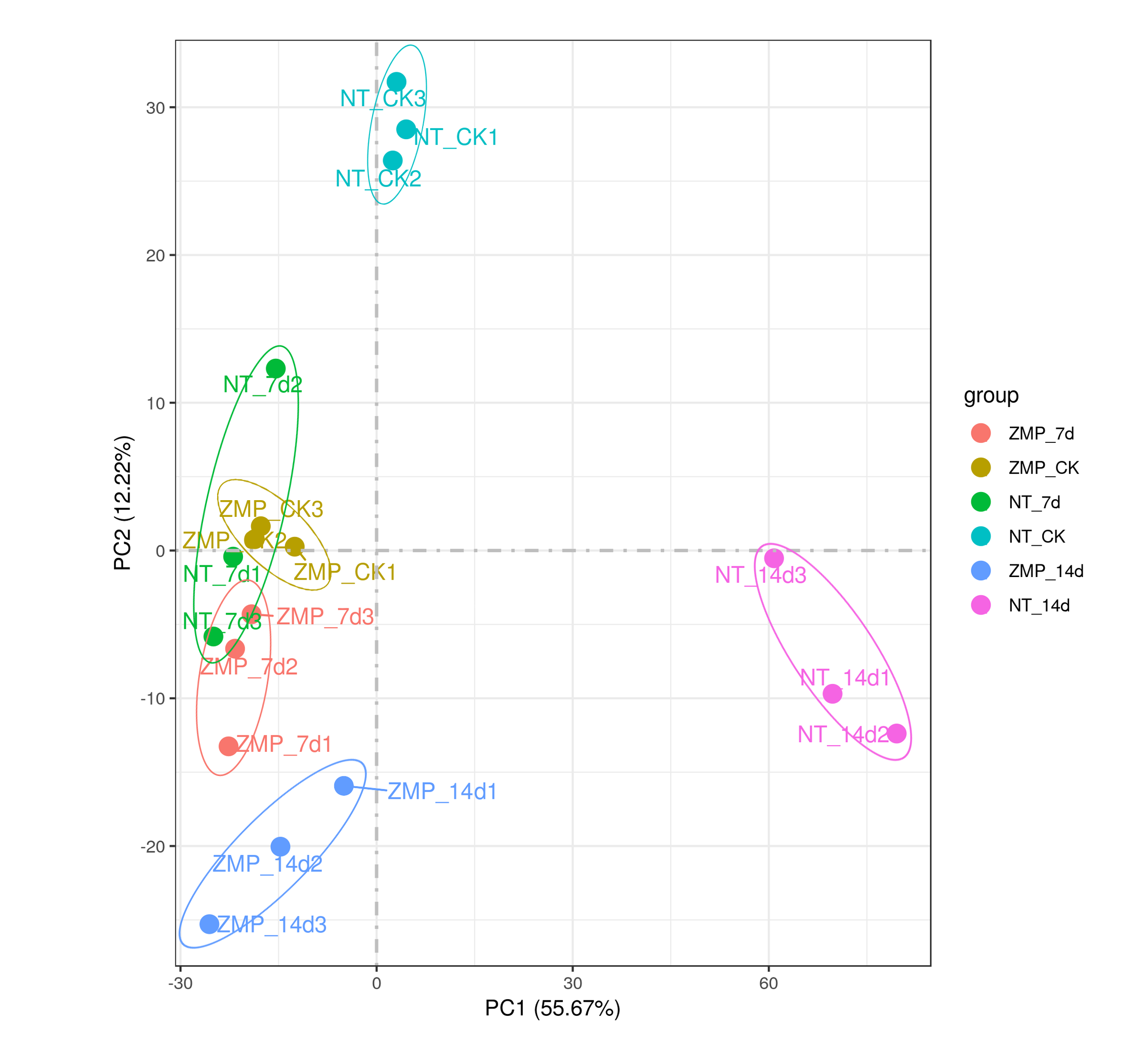

Supplement: Supplementary file 5 — Figure S5: Principal component analysis of the RNA‐Seq data. The gene expression level of all samples was analysed using PCA, represented by FPKMs (Fragments Per Kilobase of transcript per Million mapped reads). [file MPP-26-e70184-s003.tif]

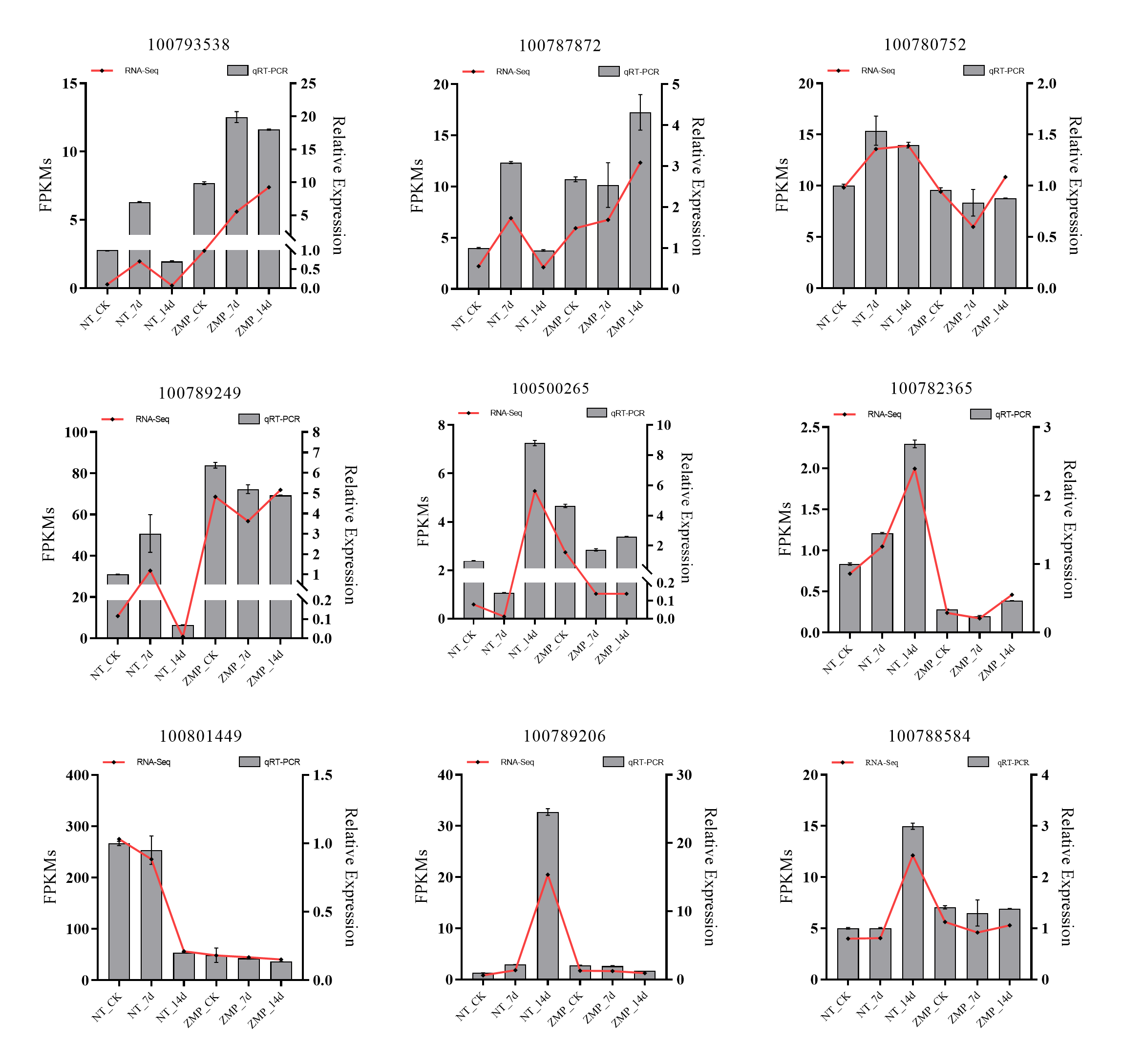

Supplement: Supplementary file 6 — Figure S6: Expression levels of nine randomly selected genes from the RNA‐Seq data. The expression patterns of genes induced by SMV in NT and ZMP plants were quantified using qRT‐PCR to validate the reliability of RNA‐Seq data. The qRT‐PCR results are presented as bar charts, while the FPKMs for each sample are displayed as lines graphs. The gene ID is annotated at the top of each subgraph. NT_CK, NT_7d and NT_14d represent the non‐transgenic plants before SMV infection, 7 and 14 days after SMV infection respectively. ZMP_CK, ZMP_7d and ZMP_14d GmMEKK2 over‐expression lines before SMV infection, 7 and 14 days after SMV infection respectively. [file MPP-26-e70184-s005.tif]

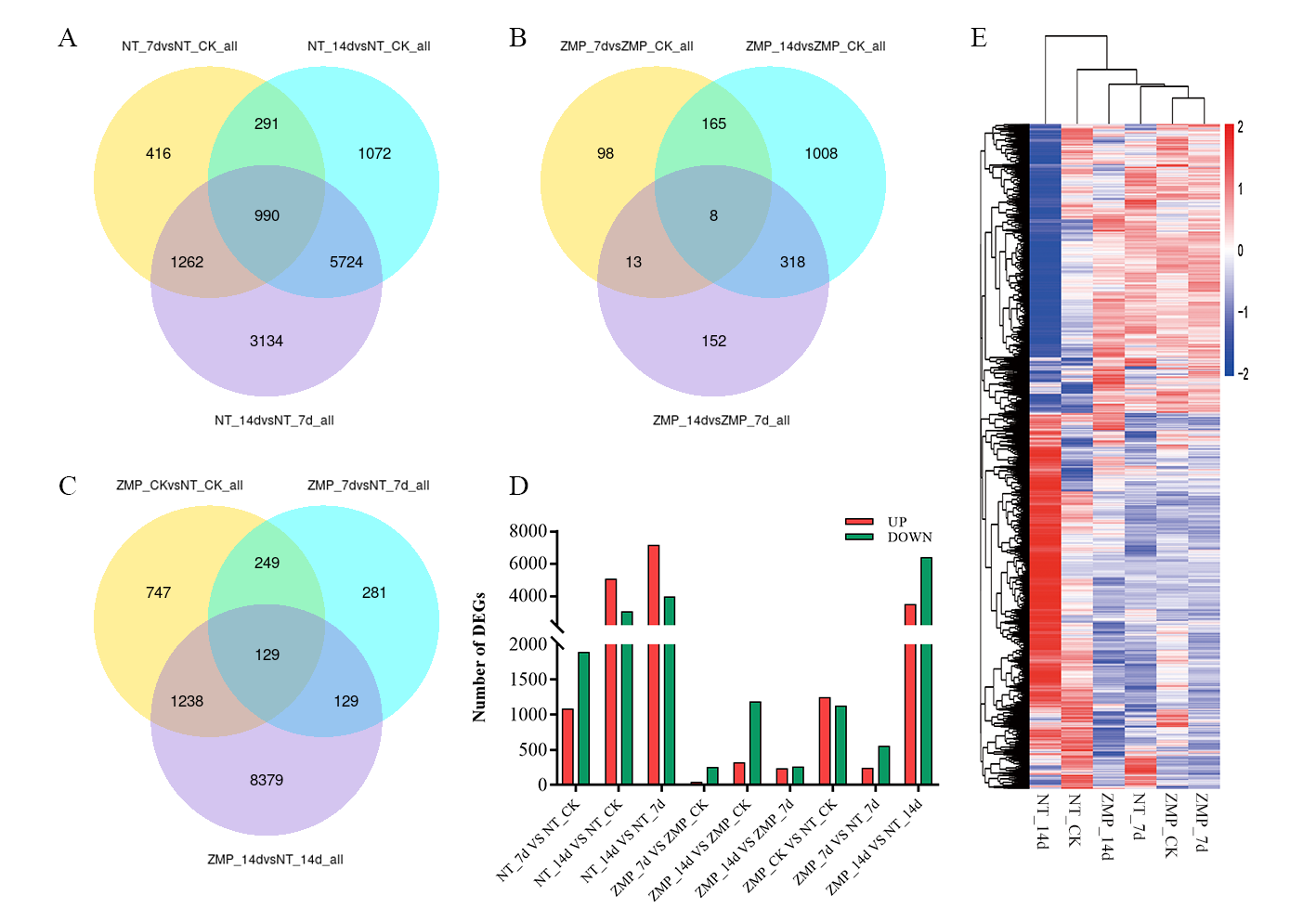

Supplement: Supplementary file 7 — Figure S7: Overview of the gene expression profile during early stages after SMV infection. (A, B) Venn diagrams of differentially expressed genes (DEGs) in NT and ZMP, respectively. (C) Venn diagrams of DEGs at each stage between NT and ZMP. (D) Counts of up‐/down‐regulated DEGs in each pairwise compared group. (E) Heatmap showing the global gene expression profile in NT and ZMP before and after SMV infection. [file MPP-26-e70184-s011.tif]

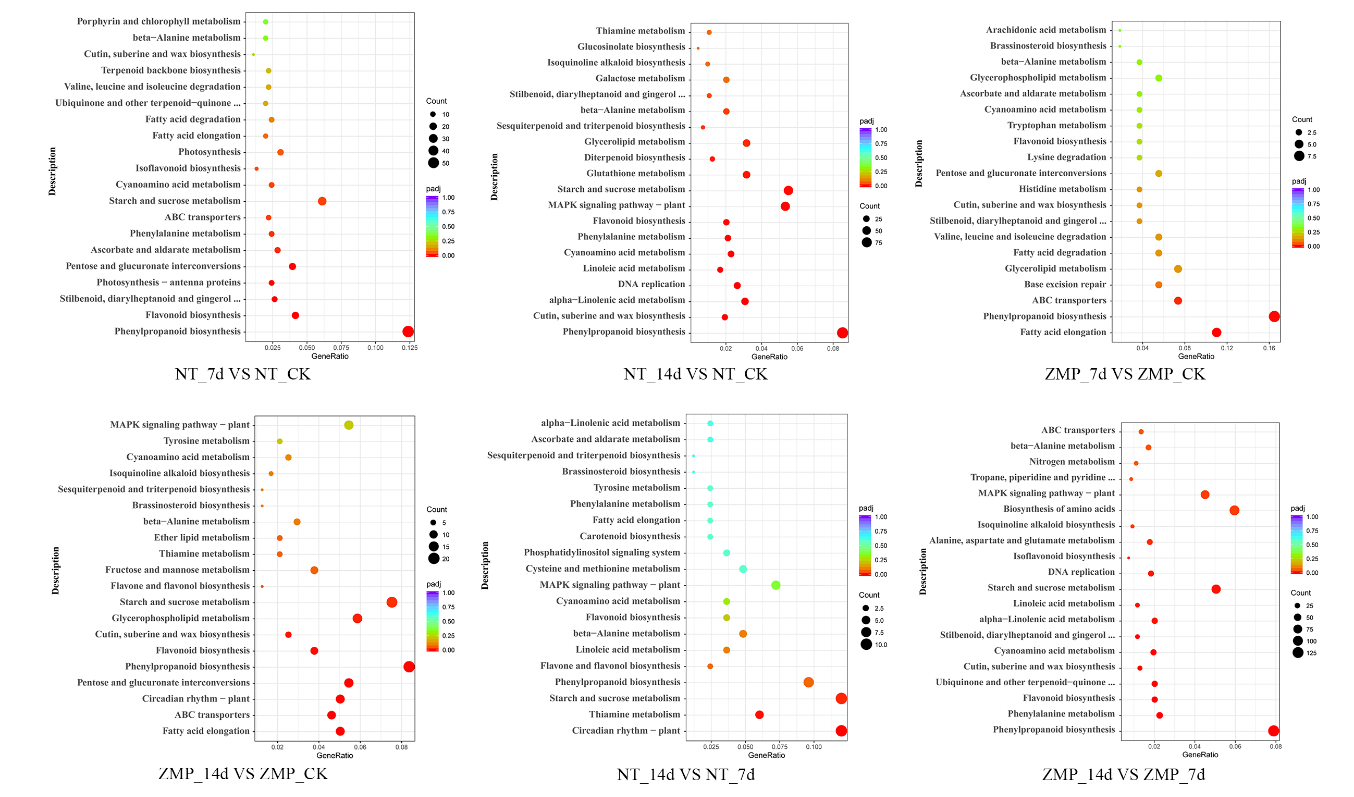

Supplement: Supplementary file 8 — Figure S8: Kyoto Encyclopedia of Genes and Genomes (KEGG) enrichment analysis of DEGs. The most significant 20 KEGG pathways in each comparison group were selected to draw scatter plots for display. The size of the dots represents the number of genes annotated to the KEGG pathway, and the colour from red to purple represents the significance of enrichment. [file MPP-26-e70184-s006.tif]
